# Biological Activity of Copper(II) and Palladium(II) Complexes with a Tetradentate S,O-Donor Ligand

**DOI:** 10.3390/ijms27135659

**Published:** 2026-06-23

**Authors:** Anita Sarić, Marina Mitrović, Ana Barjaktarević, Snežana Jovanović Stević, Biljana Petrović, Žiko Milanović, Dušan Lj. Tomović, Andriana M. Bukonjić, Djordje Petrović, Mirjana Jakovljević, Gordana P. Radić, Marina Jovanović, Irfan Ćorović, Nebojša Zdravković, Ivan Jovanović, Bojana Simović Marković

**Affiliations:** 1 Center for Molecular Medicine and Stem Cell Research, Faculty of Medical Sciences, University of Kragujevac, 34000 Kragujevac, Serbia; anitaivosevickg@gmail.com (A.S.); mitrovicmarina34@gmail.com (M.M.); ana.radovanovickg@gmail.com (A.B.); mirjana.tkb@gmail.com (M.J.); marinna034@gmail.com (M.J.); ira.corovic@gmail.com (I.Ć.); nzdravkovic@gmail.com (N.Z.); ivanjovanovic77@gmail.com (I.J.); 2 Department of Internal Medicine, Faculty of Medical Science, University of Kragujevac, Svetozara Markovića 69, 34000 Kragujevac, Serbia; 3 Department of Pharmacy, Faculty of Medical Science, University of Kragujevac, Svetozara Markovića 69, 34000 Kragujevac, Serbia; snezanaj@kg.ac.rs (S.J.S.); andriana.bukonjic@hotmail.com (A.M.B.); vasic_gordana@yahoo.com (G.P.R.); 4 Faculty of Science, University of Kragujevac, Radoja Domanovića 12, 34000 Kragujevac, Serbia; biljana.petrovic@pmf.kg.ac.rs (B.P.); djordje.petrovic@pmf.kg.ac.rs (D.P.); 5 Department of Science, Institute of Information Technologies, University of Kragujevac, Liceja Kneževine Srbije 1A, 34000 Kragujevac, Serbia; ziko.milanovic@uni.kg.ac.rs; 6 Faculty of Medicine, University of East Sarajevo, 73300 Foča, Bosnia and Herzegovina; 7 Department of Internal Medicine, General Hospital of Novi Pazar, 36300 Novi Pazar, Serbia; 8 Institute of Public Health Kragujevac, 34000 Kragujevac, Serbia

**Keywords:** Cu(II) complexes, Pd(II) complexes, thiosalicylic acid, thiopropionic acid, antitumor activity, molecular docking, DNA/BSA interactions

## Abstract

New copper(II) (C1) and palladium(II) (C2) complexes with S,O-tetradentate ligand (L) derived from thiosalicylic and thiopropionic acids were synthesized. In cell-based assays, (C1) exhibited the most pronounced activity within the tested compound series and was therefore advanced for mechanistic evaluation in 4T1 triple-negative breast cancer cells. (C1) significantly reduced 4T1 cell viability by inducing early and late apoptosis, accompanied by mitochondrial membrane depolarization and enhanced cytochrome C release. Consistently, (C1) increased the Bax/Bcl-2 ratio, promoting a pro-apoptotic shift. In parallel, (C1) triggered autophagy, as evidenced by decreased p62 and LC3B levels, induced G0/G1 cell-cycle arrest, and suppressed proliferative signaling by downregulating Ki67, cyclin D, and phosphorylated AKT. The DNA-binding studies showed moderate to strong affinity, favoring minor groove binding, with higher affinity for (C1) than for (C2). Tryptophan fluorescence quenching indicated a strong interaction with BSA via a predominantly static mechanism, more pronounced for (C1). Molecular docking at the DNA and BSA binding sites corroborated experimental findings and suggested favorable interactions between the complexes and apoptosis-related proteins (CASP3, BAX, and BCL2). The integrated experimental and computational data identify (C1) as a biologically active compound with multimodal biological effects in vitro, supporting further structural optimization and mechanistic investigation.

## 1. Introduction

Cancer remains a leading cause of death worldwide, with nearly 20 million new cases and 9.7 million deaths reported in 2022 [1,2]. The development of transition-metal-based complexes in oncology is one of the most promising areas of modern chemistry and biomedicine. Following the discovery of cisplatin, the gold standard in chemotherapy, research has increasingly focused on novel systems aimed at overcoming the limitations of conventional drugs and providing alternative mechanisms of action, such as improved selectivity, reduced systemic toxicity, and activity against resistant phenotypes [3,4]. In this context, complexes of copper(II) and palladium(II) stand out for their structural diversity, favorable biocompatibility, and potential selectivity against cancer cells [5,6,7,8]. Copper, an essential trace element involved in many vital biological processes, exhibits flexible coordination chemistry, enabling the formation of complexes with diverse geometries. This diversity directly affects their stability and reactivity at the target site of action [3]. Notably, Cu(II) complexes with thiosalicylic acid derivatives have been shown to induce apoptosis, inhibit metastasis, and exhibit pronounced cytotoxicity in cancer models [5]. Tetradentate S,O-ligands are particularly interesting, as they stabilize metal centers in biological media while preserving sufficient reactivity for productive biomolecular recognition, especially toward DNA and serum albumins [6]. Additionally, Au-Cu clusterzymes stabilized with 3-thiopropionic acid exhibit antioxidant activity comparable to that of glutathione peroxidase, catalase, and superoxide dismutase, and significantly reduce inflammatory factors in brain injury, suggesting a role in mitigating neuroinflammation [9]. Collectively, these observations emphasize the broader utility of sulfur/oxygen donor frameworks for controlling metal-centered reactivity in complex biological environments.

Although less clinically explored than platinum, palladium(II) complexes have attracted increasing attention in recent years as drug candidates, owing to their chemical similarity to Pt(II) compounds and the ligand’s amenability to modification [10]. Studies confirm the relevance of thiosalicylic and thiopropionic acid derivatives, as these ligands provide both O- and S-donor sites, enabling tetradentate coordination and remarkable stability [11,12]. A major advantage of these complexes is their ability to generate reactive oxygen species (ROS), triggering oxidative stress and apoptosis in tumor cells [13]. Furthermore, their binding to proteins such as human serum albumin enhances bioavailability and systemic transport [5,6]. This dual behavior supports multimodal mechanisms of action that combine direct and indirect cytotoxic effects.

Modern synthetic approaches to Cu and Pd complexes demonstrate that their chemical and biological properties can be fine-tuned through ligand modification [3]. Furthermore, in silico methods such as docking studies and molecular dynamics simulations of metal nanoclusters have accelerated the identification of structures with the greatest therapeutic potential [5,14,15]. Consequently, novel Cu(II) and Pd(II) complexes with thiosalicylic and thiopropionic acid derivatives represent a promising class of compounds for cancer therapy [4,5,6]. Their unique combination of stability, biomolecular interactions, and apoptosis-inducing mechanisms positions them as valuable candidates for further investigation.

In this study, a new tetradentate S,O-donor ligand derived from thiosalicylic and thiopropionic acids was synthesized together with its Cu(II) (C1) and Pd(II) (C2) complexes. The obtained compounds were structurally characterized by elemental microanalysis, IR and NMR spectroscopy, and molar conductivity measurements. The cytotoxic effects of L, C1, and C2 were evaluated by the MTT assay against mouse triple-negative breast carcinoma (4T1), human breast carcinoma (MCF-7), mouse colorectal carcinoma (CT26), and human colorectal carcinoma (HCT116) cells. Mouse mesenchymal stem cells (mMSCs) served as a non-tumor control. Given its highest antiproliferative activity and selectivity, C1 was selected for mechanistic studies in 4T1 cells. Further studies included apoptosis assessment (Annexin V/PI), mitochondrial membrane potential analysis (JC-10), cytochrome c release, autophagy evaluation (acridine orange staining and p62/LC3B profiling), cell-cycle distribution, and migration (wound-healing assay). In parallel, biomolecular interactions with calf thymus DNA and bovine serum albumin were examined by fluorescence spectroscopy. Molecular docking analyses against DNA, BSA, and apoptosis-related proteins (CASP3, BAX, and BCL2) supported the experimental findings, clarifying binding preferences and possible mechanisms of action. By integrating chemical characterization with multi-level biological readouts and structure-based modeling, this work aims to link coordination features to functional anticancer outcomes. These results provide a clearer structure–activity rationale for further optimization and preclinical exploration of S, O-tetradentate Cu(II)/Pd(II) systems.

## 2. Results and Discussion

### 2.1. Synthesis and Chemical Characterization

**Scheme 1:**
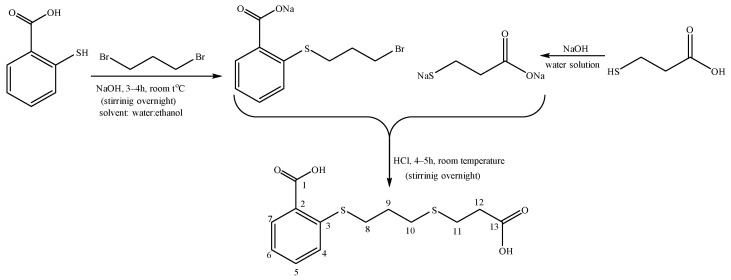
Schematic representation of the synthesis of the S,O-tetradentate ligand derived from thiosalicylic and thiopropionic acids (L).

The tetradentate S,O-ligand was synthesized from thiosalicylic acid and thiopropionic acid in a two-step procedure. In the first step, thiosalicylic acid was alkylated with 1,3-dibromopropane to form the S-propyl derivative. In the second step, this intermediate was reacted with thiopropionic acid under basic conditions to yield the final tetradentate ligand (L), as shown in Scheme 1.

**Scheme 2:**
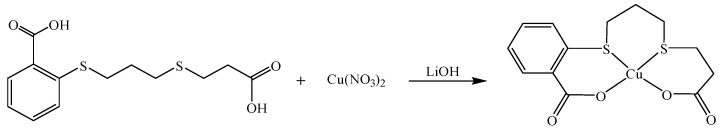
Schematic representation of the synthesis of the copper(II) complex with S,O-tetradentate ligand derivative of thiosalicylic and thipropionic acid (C1).

**Scheme 3:**
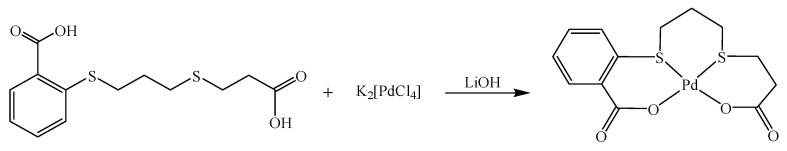
Schematic representation of the synthesis of the palladium(II) complex with S,O-tetradentate ligand, derivative of thiosalicylic and thipropionic acid (**C2**).

The corresponding copper(II) (C1) and palladium(II) (C2) complexes were prepared by direct reaction of the ligand L with Cu(NO_3_)_2_·3H_2_O or K_2_[PdCl_4_] in aqueous solution in a 1:1 metal-to-ligand molar ratio, in the presence of an equimolar amount of LiOH (Scheme 2 and Scheme 3).

The composition of the synthesized compounds was confirmed by elemental microanalysis, indicating that the experimentally obtained values are in good agreement with the theoretically calculated percentages of carbon, hydrogen, and sulfur. Furthermore, based on the mass spectrometry results, it can be concluded that the obtained masses for the S,O-tetradentate ligand and the corresponding copper(II) and palladium(II) complexes are consistent with the expected values (323.0382 for the ligand, 362.9785 for the copper(II) complex, and 404.9446 for the palladium(II) complex), with only minimal and acceptable deviations observed (mass error: 0.15 ppm for the ligand, 0.00 ppm for the copper(II) complex, and 1.16 ppm for the palladium(II) complex) (Supplementary Figure S1).

The IR spectra of the ligand and its copper(II) and palladium(II) complexes were recorded using the KBr pellet technique. The obtained spectra confirmed the successful formation of the tetradentate S,O-ligand and its corresponding metal complexes. The spectra exhibited characteristic absorption bands consistent with the proposed structures (Table 1). The most diagnostic features were the bands associated with the asymmetric stretching vibrations of the carboxylate groups, which clearly indicated coordination of the metal ions to the S,O-tetradentate ligand. In accordance with previously reported data for structurally related systems [16,17,18], the asymmetric stretching frequencies of the carboxylate groups provide a reliable criterion for differentiating between protonated (1700–1750 cm^−1^) and coordinated (1600–1700 cm^−1^) carboxylate functionalities [19].

The IR spectrum of the copper(II) complex with the tetradentate S,O-ligand derived from thiosalicylic and thiopropionic acids exhibits characteristic spectral changes consistent with coordination through both oxygen and sulfur donor atoms. In the free ligand (L), the asymmetric stretching vibration of the carboxylate group appears at 1672 cm^−1^, which is slightly lower than typical values due to the presence of adjacent R–S groups. Upon complexation, these bands shift to 1614 and 1588 cm^−1^ for complex C1 and to 1581 and 1553 cm^−1^ for complex C2, confirming deprotonation and coordination of the carboxylate moieties to the metal center [20,21]. Coordination through sulfur is further supported by shifts in the C–S stretching bands. In the free ligand, this vibration is observed in the range 650–750 cm^−1^ (centered at 740 cm^−1^), whereas in the complexes it appears in the range 620–700 cm^−1^ (746 cm^−1^ for C1 and 745 cm^−1^ for C2), indicating moderate metal–sulfur interaction.

The chemical shifts of hydrogen and carbon atoms in the NMR spectra of the S,O-tetradentate ligand and its palladium(II) complexes were observed at the expected positions and were largely consistent with those of the free ligand. Minor differences were detected in the chemical shifts of the carboxylate carbon signals, appearing at 167.7 and 175.28 ppm for the ligand and at 167.72 and 173.27 ppm for the palladium(II) complex. These small shifts indicate that the carboxyl groups are protonated in the free ligand and become deprotonated upon complex formation, supporting coordination of the metal ions through the oxygen atoms of the carboxylate groups. This behavior is consistant with literature data for structurally related copper(II)-complexes with S,O-donor ligands, where coordination through sulfur results in pronounced shifts of the thiocarbonyl carbons, while aliphatic carbons exihibit only minor changes due to their distance from the coordination centar [6].

Molar conductivity measurements were carried out in DMSO solutions of the synthesized copper(II) and palladium(II) complexes at a concentration of 1 × 10^−3^ mol L^−1^. The obtained molar conductivity values, all below 50 S cm^2^ mol^−1^, indicate that the complexes behave as electrically neutral species in solution.

### 2.2. The Tested Complexes Showed Dose-Dependent Cytotoxicity

**Figure 1:**
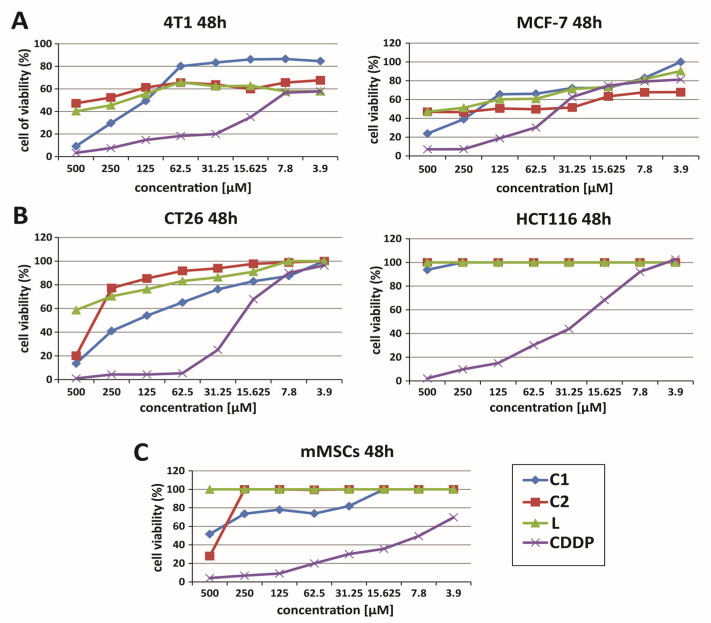
Dose-dependent cytotoxicity of C1 and C2 complexes. The graphs depict the survival rates of (**A**) murine breast cancer (4T1) and human breast carcinoma (MCF-7) cells, (**B**) murine colorectal carcinoma (CT26) and human colorectal carcinoma (HCT116) cells, and (**C**) mouse mesenchymal stem cells (mMSCs) following 48 h of incubation with complexes C1 and C2, the free ligand L, and cisplatin (CDDP), as determined by the MTT assay. All data are presented as mean values from three independent experiments performed in triplicate.

The effects of the investigated compounds on the viability of several tumor cell lines (4T1, CT26, MCF-7, and HCT116), as well as non-tumor mouse mesenchymal stem cells (mMSCs), were evaluated using the MTT assay. The cells were treated with the compounds for 48 h at concentrations ranging from 3.9 to 500 μM. The MTT assay analysis demonstrated that all tested compounds reduced the viability of murine (4T1) and human (MCF-7) breast cancer cells, as well as mouse colorectal carcinoma cells (CT26) (Figure 1A,B).

All tested compounds exhibited dose-dependent cytotoxic activity against breast cancer cell lines (Figure 1A) and low cytotoxicity toward mMSCs (Figure 1C). Notably, none of the compounds showed cytotoxic effects on the human colorectal cancer cell line HCT116, in contrast to their activity against the murine counterpart CT26 (Figure 1B). Importantly, complex C1 exhibited significantly higher cytotoxic activity against both murine and human breast cancer cell lines (4T1 and MCF-7), as well as murine colorectal cancer cells (CT26), compared with complex C2 and the free ligand L (Figure 1A,B).

At higher concentrations (250–500 μM), the cytotoxic effects of C1 were comparable across all tested cancer cell lines, except for HCT116 cells. In contrast, at intermediate concentrations (62.5–125 μM), C1 exhibited greater cytotoxicity against 4T1 cells compared with C2 and the free ligand L. At lower concentrations (3.9–31.25 μM), the cytotoxic effects of C1 were similar to those of the other tested compounds across all examined cell lines.

Table 2 summarizes the IC_50_ values of the tested compounds against murine 4T1 and CT26 cells, as well as human MCF-7 and HCT116 cancer cell lines. Based on these values, complex C1 exhibited higher cytotoxic activity against murine 4T1 and CT26 cells compared with the other tested compounds. Notably, the highest cytotoxic effect of C1 was observed against murine 4T1 tumor cells (IC_50_ = 146.22 ± 28.23 μM), which led to its selection for further analysis within the studied compound series. Although its IC_50_ values are higher than those of cisplatin, C1 demonstrated the most consistent activity and moderate selectivity among the tested compounds, which justified its selection for mechanistic studies (to further assess reproducibility, additional independent MTT experiments were performed for 4T1 and CT26 cells, yielding highly comparable IC_50_ values and confirming the consistency of C1 cytotoxic activity, see Supplementary Figure S2). In contrast, complex C2 and the free ligand L demonstrated higher cytotoxic activity toward human MCF-7 cancer cells (Table 2). Overall, the results indicate that all tested compounds reduce cancer cell viability in a dose-dependent manner, suggesting that the newly synthesized complexes possess potential as anticancer agents, particularly against breast cancer. However, it should be emphasized that the cytotoxic potency of all tested complexes, including C1, remains significantly lower than that of cisplatin (CDDP), indicating that their activity should be interpreted within the framework of preliminary in vitro evaluation.

To assess potential side effects, including cytotoxicity toward healthy cells, all compounds and cisplatin were evaluated against non-tumor mMSCs (Figure 1C). All tested compounds reduced the viability of mMSCs in a dose-dependent manner.

Cisplatin also exhibited higher cytotoxic effects toward mMSCs compared with the investigated Cu(II) and Pd(II) complexes (Table 2). Complex C1 demonstrated the highest degree of cytotoxic selectivity, as indicated by the selectivity index (IC_50_ mMSCs/IC_50_ tumor cells) (Table 3), and was therefore selected for all subsequent analyses.

### 2.3. Induction of Apoptotic Cell Death in 4T1 Cells Treated with Newly Synthesized Cu(II) Complex

**Figure 2:**
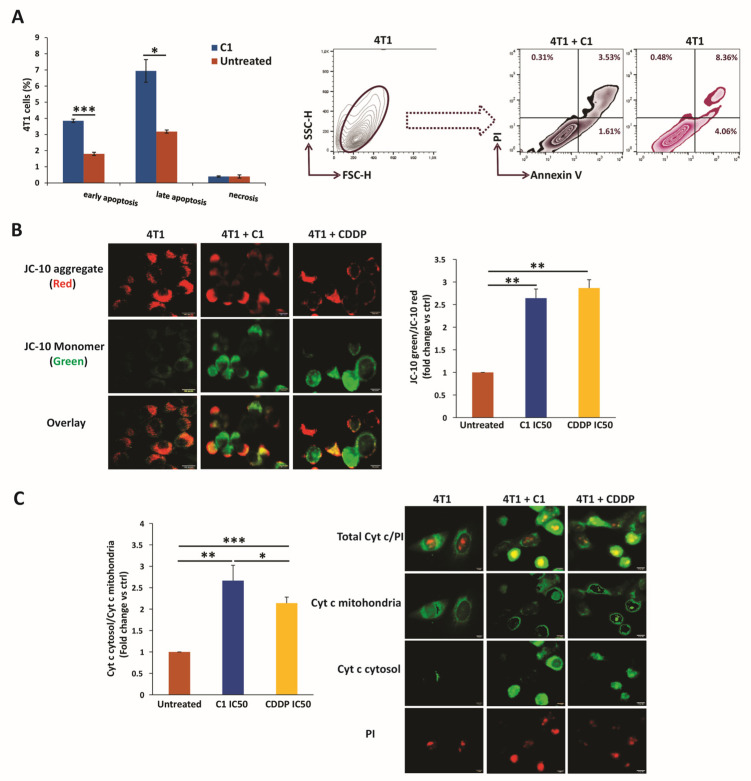
C1 promotes apoptotic cell death in 4T1 cancer cells. Apoptosis of untreated and C1 complex-treated 4T1 cells were evaluated by flow cytometry using Annexin V (FITC) and propidium iodide (PI) double staining. Representative FACS plots present the population of viable (AnnV- PI−), early apoptotic (AnnV+ PI−), late apoptotic (AnnV+ PI+), and necrotic (AnnV- PI+) 4T1 cells (**A**). After 48 h of treatment with C1 and CDDP at their IC50 concentrations, 4T1 cells were stained using JC-10. The green fluorescence in the cytoplasm from JC-10 monomers increased compared to the control group, while the red fluorescence from mitochondrial aggregates decreased. The histogram bars ± SEM show the mean fold change in the JC-10 green/red ratio in cells (**B**). After 48 h, both untreated and C1 and CDDP-treated 4T1 cells exhibited immunolocalization of cytochrome c, showing green punctate staining in the mitochondria and green diffuse staining in the cytoplasm. PI red fluorescence increased in dead cells. A bar graph shows the fold-change ratio of cytosolic to mitochondrial cytochrome c (**C**). Bars = mean ± SEM. * *p* < 0.05, ** *p* < 0.01, *** *p* < 0.001 vs. control group.

Apoptosis, also known as programmed cell death, is characterized by a specific cascade of biochemical events, including DNA fragmentation, that leads to cell death [22]. The potential of investigated complexes to induce apoptotic death was determined by flow cytometry of both untreated and treated cells after double staining with Annexin V FITC and propidium iodide. All tested complexes at the tested concentration induced apoptotic death of 4T1 cells. Figure 2A shows that the majority of apoptotic 4T1 cells were in late apoptosis, 24 h after the treatment with C1. However, we did not observe a statistically significant difference in the percentage of necrotic cells compared to the control group (Figure 2A). Finally, our findings show that the C1 complex induced apoptotic cell death in mouse breast cancer cells after 24 h.

### 2.4. C1 Induces Alterations in Mitochondrial Membrane Potential (ΔΨM) and Facilitates the Cytochrome c Release

Different antitumor agents can trigger apoptosis by activating the intrinsic mitochondrial pathway [23]. The death signals from both intrinsic and extrinsic apoptotic pathways are integrated by mitochondria. The imbalance of mitochondrial membrane potential (ΔΨM) and the release of cytochrome c from mitochondria are important factors that control mitochondrial apoptosis. We assessed the capacity of C1 to induce mitochondrial apoptosis in 4T1 cells by analyzing changes in ΔΨM using the fluorescent dye JC-10. In living cells, JC-10 primarily generates red fluorescent aggregates in the mitochondrial matrix. In dying cells, the JC-10 primarily diffuses from the mitochondria into the cytoplasm, resulting in the formation of a green fluorescent monomeric form. Treatment with C1 resulted in increased green fluorescence and diminished red fluorescence, indicating a loss of ΔΨM. When compared to untreated cells, treatment of 4T1 cancer cells with C1 led to a significant increase (** *p* = 0.007) in the green-to-red JC-10 ratio, which rose by 2.6 times. This effect was similar to that observed with CDDP, which resulted in a 2.8-fold decrease in ΔΨM (Figure 2B). To further assess C1’s potential role in inducing mitochondrial apoptosis, we investigated the cellular distribution of cytochrome c in 4T1 cells treated with C1, utilizing immunofluorescence. In untreated 4T1 cells, cytochrome c exhibited a punctate pattern with increased green intensity, which is consistent with its localization in the mitochondria. The C1-treated 4T1 cells demonstrated a significant release of cytochrome c (** *p* = 0.001) from the mitochondria into the cytosol, exhibiting an increase of 2.7-fold compared to the control. Moreover, C1 resulted in a greater release of cytochrome c (* *p* = 0.041) compared to CDDP in 4T1 cells, despite CDDP causing a 2.2-fold increase (Figure 2C). This finding indicates that C1 may induce mitochondrial apoptosis by disrupting ΔΨM and promoting the release of cytochrome c, which results in cell death and surpasses the effects of CDDP.

**Figure 3:**
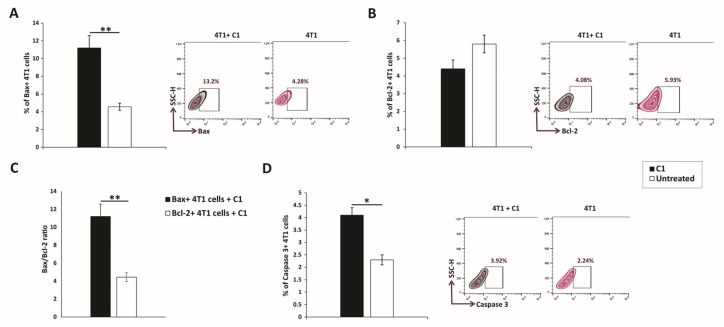
The C1 complex induced apoptosis of murine breast cancer cells. Expression of key apoptosis-related molecules in tumor cells after C1 complex treatment. Expression of Bax and Bcl-2 proteins as well as caspase 3 was evaluated by flow cytometry. The expression of Bax and Bcl-2 proteins as well as caspase 3 expression with representative FACS dot plots in group treated with C1 for 24 h and untreated (**A**,**B**,**D**). Ratio of proapoptotic and antiapoptotic molecules (**C**). The data are presented as means ± SEM (standard error of the mean) of three independent experiments; * *p* < 0.01, ** *p* < 0.001 indicates a statistically significant difference between treated and untreated cells.

An imbalance between proapoptotic proteins (such as Bax) and antiapoptotic proteins (such as Bcl-2), known as the protectors of the mitochondrial membrane, triggers the intrinsic or mitochondrial apoptotic pathway. This imbalance affects the permeability of the mitochondrial membrane, causes cytochrome c release from mitochondria, and activates caspase-3, which consequently leads to apoptosis [24]. After treating cells with IC_50_ concentrations of C1 complex, there was a significant increase in the percentage of Bax+ 4T1 cells (Figure 3A). In line with these results, C1 complex significantly decreased the percentage of Bcl-2+ 4T1 cells (Figure 3B). Moreover, an increase in the Bax/Bcl-2 ratio that implicates predominance of pro-apoptotic molecules in treated 4T1 cells emphasizes the involvement of C1 in promoting apoptotic signaling (Figure 3C). The C1 complex increased the percentage of caspase-3+ cells in 4T1 cancer cells, but it did not reach statistical significance (Figure 3D). In line with these data, C1 induces mitochondrial apoptosis by disrupting ΔΨM and promoting the release of cytochrome c, which further results in apoptotic cell death.

### 2.5. C1 Induces Autophagy and Downregulates p62 and LC3B in 4T1 Cells

**Figure 4:**
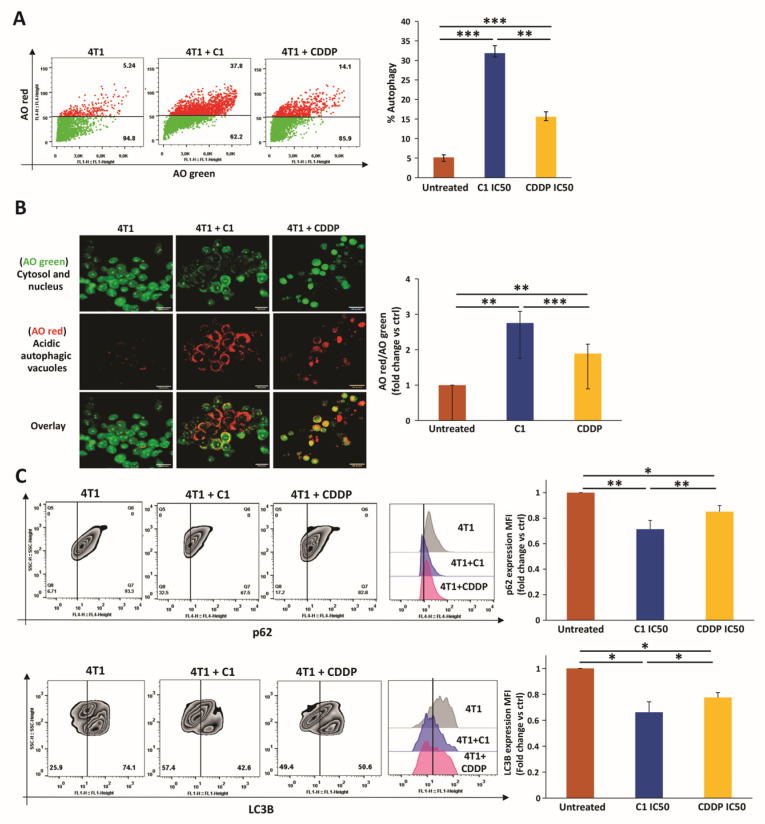
C1 induces autophagy and downregulates p62 and LC3B in 4T1 cells. (**A**) Cells were treated for 48 h with C1 and CDDP at their respective IC_50_ values and then stained with AO. The percentage of cells generating AO-red or AO-green fluorescence was measured by flow cytometry. Bar graphs showed the mean % autophagy ± SEM (n = 3). ** *p* < 0.01 and *** *p* < 0.001 indicate significance compared to the control. (**B**) AO staining indicated autophagic vacuoles in 4T1 cells treated with C1. In AO staining, autophagic acidic vacuoles appear bright red or orange-red, and the nucleus and cytoplasm are green. The bar graphs show the mean fold change in MFI for AO red/AO green ± SEM. Statistical significance: ** *p* < 0.01 and *** *p* < 0.001 vs. control. (**C**) Flow cytometry was used to measure the MFI of the autophagic markers p62 and LC3B in C1 and CDDP-treated 4T1 cells. The data are presented as histogram bars, illustrating the mean fold change in MFI for p62 and LC3B ± SEM. * *p* < 0.05 and ** *p* < 0.01 indicate statistical significance relative to the control group. (Scale bars = 100 µm; Magnification 40×).

Numerous stress signals, mitochondrial dysregulation, and antitumor drugs may induce autophagy [25]. Although autophagy is acknowledged as a process that supports cell survival, its overactivation can result in cell death. Consequently, we conducted a flow cytometry analysis and acridine orange (AO) labeling to investigate the influence of C1 on the regulation of autophagy in 4T1 cells. Autophagic cells enable the entry of AO into lysosomes and acidic compartments, leading to protonation and the emission of red fluorescence, while non-autophagic cells produce green fluorescence. Treatment with C1 (*** *p* < 0.001) and CDDP (** *p* = 0.003) significantly increased the percentage of autophagic 4T1 cells to 31.9% and 15.6%, respectively, compared to the control. Furthermore, C1 demonstrated greater efficiency in enhancing autophagy (** *p* = 0.001) in comparison to the effect of CDDP (Figure 4A). Fluorescence microscopy analysis confirmed the presence of autophagic vacuoles, showing that cells treated with C1 (** *p* = 0.001) and CDDP (** *p* = 0.006) exhibited a significant increase in red AO fluorescence by 2.7- and 1.9-fold compared to the control group, which mainly displayed AO green fluorescence (Figure 4B). Furthermore, treatment with C1 and CDDP resulted in a reduction in autophagic flux markers, with p62 decreasing by 0.71- and 0.83-fold and LC3B by 0.66- and 0.78-fold, respectively, when compared to untreated 4T1 cells (Figure 4C). Notably, C1 demonstrated more efficient inhibition of both p62 (** *p* = 0.003) and LC3B (* *p* = 0.014) expressions when compared to the effect of CDDP. The findings suggest that C1 could have a crucial role in activating autophagy in 4T1 breast cancer cells, exceeding the impact of CDDP.

### 2.6. C1 Induces G0/G1 Cell Cycle Arrest in 4T1 Cells

**Figure 5:**
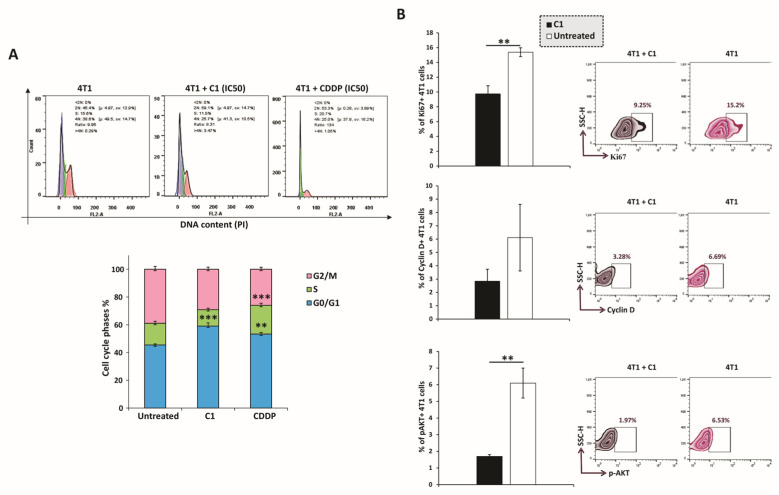
The C1 complex attenuates Ki67 expression in 4T1 tumor cells. The graphs show the mean percentage of tumor cells ± SEM in different phases of the cell cycle in 4T1-treated versus untreated cells (**A**). The representative FACS plots present expression of Ki67 in 4T1 cells exposed to C1 complex for 24 h and untreated 4T1 cells determined by flow cytometry (**B**). The C1 complex influences the expression of cell cycle regulators in 4T1 cells. The percentage of cyclin D positive cells treated versus untreated (**B**). The C1 complex regulates cell cycle decreasing p-AKT expression in 4T1 cells. Analysis of p-AKT expression in 4T1 cells after C1 complex treatment at a concentration of IC_50_ compared to untreated 4T1 cells for 24 h was determined by flow cytometry. Percentage of p-AKT+ 4T1-treated versus untreated cells (**B**). The data are presented as means ± SEM (standard error of the mean) of three independent experiments; ** *p* < 0.01, *** *p* < 0.001 indicates a statistically significant difference between treated and untreated cells.

DNA damage and disturbed cell cycle regulation are often associated with tumor cell apoptosis. We utilized flow cytometry to examine the distribution of propidium iodide (PI)-stained C1-treated 4T1 cells throughout the cell cycle and compared it to CDDP-treated and untreated 4T1 cells. The results showed a significant increase (*** *p* < 0.001) in the number of C1-treated 4T1 cells in the G0/G1 phase, reaching 59.1%, compared to 53.1% in CDDP-treated cells and 45.4% in untreated cells (Figure 5A). This finding suggests that C1 may successfully induce G0/G1 cell cycle arrest in cancer cells, which may contribute to C1’s induction of apoptosis. Moreover, antiproliferative effects of C1 were evaluated by the assessment of Ki67 expressing 4T1 treated cells. Ki67 is a DNA-binding protein expressed throughout the cell cycle (in proliferating but not in quiescent cells) [26]. As it is shown in Figure 5B, C1 significantly reduced the percentage of Ki67 positive 4T1 cells. Cyclin D, an oncogene that facilitates progression through the G1 phase via interactions with cyclin-dependent kinases Cdk4 and Cdk6, and cyclin E, which governs the G1/S transition through its interaction with Cdk220 [27], were downregulated following C1 treatment (Figure 5B). These findings highlight a potential mechanism by which C1 suppresses cell cycle progression and inhibits tumor cell growth. Finally, we analyzed the impact of C1 on the PI3K/AKT/mTOR signaling pathway, a key regulator of tumor cell growth, proliferation, and survival [28]. Using flow cytometry, we observed a significant reduction in the percentage of p-AKT-positive 4T1 cells treated with C1 compared to untreated cells (Figure 5B). This reduction in AKT activity likely contributed to the inhibition of proliferation and decreased survival of tumor cells.

### 2.7. Effect of C1 Treatment on Wound Closure in 4T1 Cells

**Figure 6:**
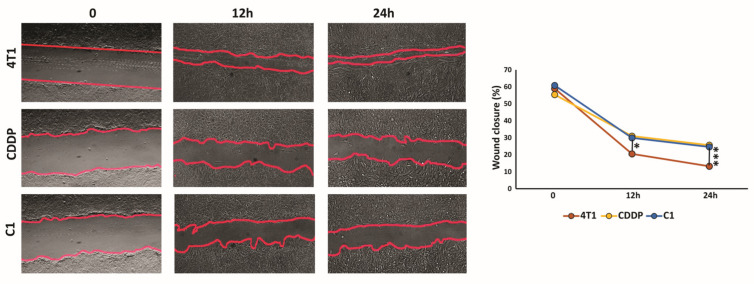
Effect of C1 treatment on wound closure in 4T1 cells. In a wound-healing experiment, C1 reduced the migration of 4T1 cells. The images depict scratched and wound-healing regions (marked by red dotted lines) on monolayers of 4T1 cancer cells at various time points (0, 12, and 24 h) with IC_50_ (μM) of C1 treatment. The wound healing assay was used to quantify the migration area in 4T1 cells treated with C1, cisplatin, or non-treated cells after 0 h, 12 h, and 24 h. Migratory and invasive cells in over five microscopic fields. The migration rates of 4T1 cells treated with C1, cisplatin, or control (untreated 4T1 cells) after 0 h, 12 h, and 24 h. Additional wound-healing experiments performed using a sub-cytotoxic concentration of C1 (IC_20_ range; >90% cell viability) did not demonstrate statistically significant differences in wound closure compared with untreated controls and are presented in Supplementary Figure S3. The data are presented as means ± SEM (standard error of the mean) of three independent experiments; * *p* < 0.1, *** *p* < 0.001 indicates a statistically significant difference between treated and untreated cells.

Cell migration occurs during the whole cascade of cancer development and is especially important during invasion, which is the initial step of metastasis [29]. The effect of C1 on cell migration was assessed by performing a scratch assay (wound healing assay). Treatment with C1 at IC_50_ concentrations resulted in significantly reduced wound closure in 4T1 cells compared with untreated controls, while cisplatin produced a comparable effect (Figure 6). Quantification of wound closure using ImageJ software (version 1.53e, National Institute of Health, Bethesda, MD, USA) demonstrated significantly decreased closure at both 12 h and 24 h following C1 treatment. To further distinguish between effects on cell motility and potential confounding effects of cytotoxicity or proliferation inhibition, additional wound-healing experiments were performed using a sub-cytotoxic concentration of C1 (IC_20_ range), selected to maintain cell viability above 90%. Under these conditions, no statistically significant differences in wound closure were observed between C1-treated and untreated cells at either 12 h or 24 h (Supplementary Figure S3). These findings suggest that the reduced wound closure observed at IC_50_ concentrations is likely influenced, at least in part, by the antiproliferative and cytotoxic effects of C1. Nevertheless, given the concentration-dependent nature of anticancer drug responses, a potential contribution of higher C1 concentrations to impaired wound closure cannot be completely excluded.

**Figure 7:**
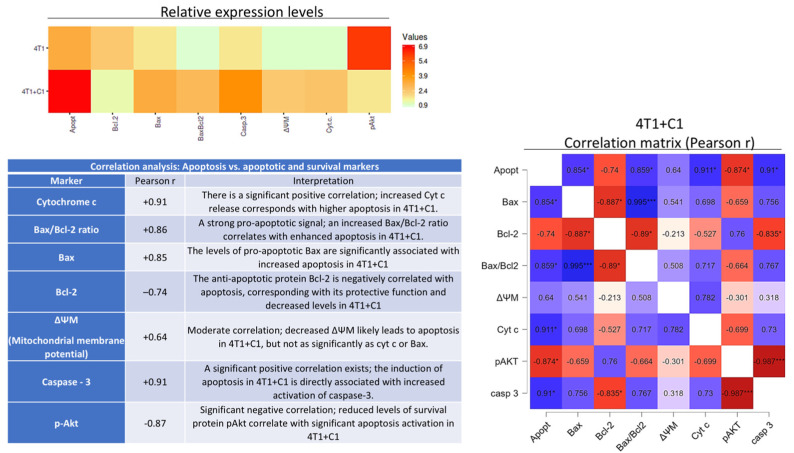
C1 significantly modulates apoptosis. Heatmap showing the fold change in apoptosis and expressions of key related molecules following C1 (IC_50_) treatment of 4T1 cells for 24 h, compared to the control. A qualitative correlation table illustrating the relationships between apoptosis and its key protein markers following C treatment of 4T1 cells. * *p* < 0.01, *** *p* < 0.001.

In line with this interpretation, our findings clearly demonstrated that C1 induced apoptosis, regulated autophagy, and altered cell cycle progression, thereby further strengthening the evidence for the anticancer activity of newly synthesized Cu(II) complexes (Figure 7). Several limitations of the present study should be acknowledged. Although additional independent experiments confirmed the reproducibility of C1 cytotoxic activity (Supplementary Figure S2), variability in IC_50_ determination represents an inherent feature of cell-based assays and may reflect biological heterogeneity and technical variability. Therefore, the observed cytotoxic activity should be interpreted within the framework of preliminary in vitro evaluation. Moreover, while the mechanistic findings support the biological activity of C1, additional validation in expanded experimental systems and in vivo tumor models will be required to further establish its therapeutic relevance. Taken together, our findings identify C1 as the most biologically active complex within the tested series, capable of inducing apoptosis, modulating autophagy, and suppressing proliferation potential in 4T1 cells.

### 2.8. Computational Study of C1 Binding to Pro- and Anti-Apoptotic Proteins

Molecular docking simulations of **C1** within the active sites of CASP3, BCL2, and BAX revealed distinct differences in binding affinities and stabilization mechanisms, as summarized in Table 4. The strongest interaction was observed for CASP3, with a binding free energy of ΔG_bind_ = –7.17 kcal/mol and a K_i_ of 5.53 µM, indicating that C1 binds tightly within the catalytic site of caspase-3. However, such binding should not be interpreted as direct enzymatic activation, since occupation of the catalytic pocket of caspase-3 is more commonly associated with enzyme inhibition rather than activation. Such interaction may facilitate caspase activation, thereby promoting apoptosis through the execution phase of the intrinsic pathway.

For BCL2 and BAX, the obtained binding energies were less favorable (Δ*G*_bind_ = –5.88 kcal/mol, Ki = 49.25 µM, and ΔG_bind_ = –6.01 kcal/mol, K_i_ = 39.36 µM, respectively). The relatively weak binding affinity toward BCL2, an anti-apoptotic protein, suggests that C2 does not stabilize this complex, which aligns with the experimentally observed reduction in BCL2^+^ cells after C2 treatment. Conversely, the moderate interaction with BAX implies that C1 may assist in promoting the pro-apoptotic conformation of this protein, facilitating mitochondrial membrane permeabilization and the subsequent release of cytochrome c.

Taken together with our biological data, these results suggest that C1 primarily acts upstream of CASP3 by inducing mitochondrial dysfunction (loss of ΔΨM and cytochrome c release), which subsequently leads to proteolytic activation of CASP3. Pharmacological inhibition of CASP3 significantly reduced cell death, confirming that CASP3 activity is required for the execution of apoptosis. Therefore, the interaction of C1 with CASP3 should be considered a potential off-target or modulatory effect rather than the primary mechanism of apoptosis induction.

Theoretical findings are consistent with experimental observations, where treatment with the C1 complex led to a significant increase in BAX^+^ cells and a decrease in BCL2^+^ cells, resulting in an elevated BAX/BCL2 ratio, a hallmark of mitochondrial apoptosis activation. Although the increase in CASP3^+^ cells was not statistically significant, the trend corresponds to docking predictions, indicating that C1 can interact favorably with the caspase-3 active site.

Overall, the combined computational and experimental results support a mechanism in which C1 induces mitochondrial (intrinsic) apoptosis by destabilizing anti-apoptotic BCL2, enhancing BAX activation, and facilitating CASP3 interaction. The negative ΔG_bind_ values confirm that these interactions are spontaneous and energetically favorable, emphasizing C2’s potential as a pro-apoptotic and antitumor agent.

**Figure 8:**
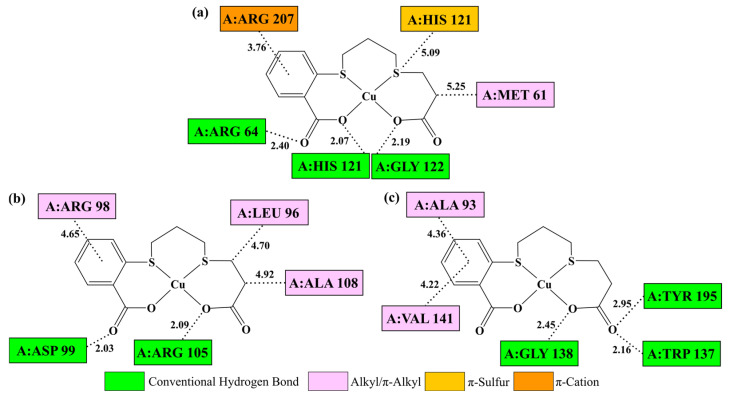
The important interactions of C1 in the (**a**) caspase 3 (CASP3), (**b**) anti-apoptotic B-cell lymphoma 2 (BCL2) enzyme, and (**c**) Bcl-2-associated X protein (BAX). For better clarity, the remaining part of the protein structure is excluded. Different colours represent different types of interactions (legend).

The molecular docking analysis of C1 with CASP3, BCL2, and BAX revealed distinct interaction profiles that explain its differential binding affinities and possible mechanistic roles in apoptosis regulation, as shown in Figure 8. For CASP3, C1 displayed a strong and well-defined interaction pattern characterized by multiple hydrogen bonds with residues A:ARG64, A:HIS121, and A:GLY122. Additionally, π–sulfur and hydrophobic contacts with A:ARG207 and A:MET61 further stabilized the complex. These interactions likely facilitate favorable positioning of C1 within the active pocket, supporting its predicted role in promoting CASP3 activation, which is consistent with theoretical and experimental findings that indicated increased CASP3^+^ cell populations after treatment.

In the case of BCL2, the C1 complex predominantly interacted via hydrogen bonds with A:ASP99 and A:ARG105, complemented by alkyl and π–alkyl interactions with A:LEU96 and A:ALA108. The presence of these non-polar contacts reflects the hydrophobic nature of the BCL2 binding pocket and suggests that C1 weakly stabilizes this anti-apoptotic protein. This observation correlates with the experimental reduction in BCL2^+^ cells, implying that C1 binding may contribute to BCL2 functional inhibition and destabilization.

For BAX, C1 engaged in several polar interactions with A:GLY138, A:TYR195, and A:TRP137, along with π–sulfur and alkyl interactions with A:VAL141 and A:ALA93. These interactions indicate strong stabilization within the hydrophobic core of the BAX structure, potentially facilitating its pro-apoptotic activation through conformational rearrangement.

Overall, the docking results demonstrate that C1 forms stable complexes with CASP3, BCL2, and BAX, mediated by both polar and hydrophobic forces. The interaction patterns suggest a dual apoptotic modulation mechanism, in which C1 enhances the activation of CASP3 and BAX, while simultaneously interfering with the stability of BCL2. These findings are fully consistent with the experimental evidence showing increased BAX/BCL2 ratio and elevated CASP3 activity, supporting the proposed role of C1 as an effective pro-apoptotic agent capable of triggering mitochondrial-mediated cell death. These findings are fully consistent with the experimental evidence showing increased BAX/BCL2 ratio and CASP3 involvement in C1-induced mitochondrial apoptosis.

### 2.9. Interactions with Biomacromolecules

#### DNA Binding Studies

The study of DNA interactions with metal complexes is of great importance in bioinorganic and pharmaceutical research, as these compounds hold potential in the development of new therapeutic agents. Transition metal complexes, in particular, display diverse coordination geometries and electronic characteristics, which allow them to associate with DNA through various mechanisms, possibly leading to structural or functional modifications of the nucleic acid. Such interactions are relevant for elucidating the molecular basis of activity for numerous metal-containing drugs, including those with anticancer and antimicrobial properties. These complexes may interact with DNA via several non-covalent binding modes, such as intercalation between base pairs, groove association (within the major or minor groove), or electrostatic interactions involving oppositely charged regions. Intercalative binding generally involves planar aromatic systems that insert between base pairs and stabilize the complex through π–π interactions. Groove binding depends on the spatial compatibility of the complex with the DNA helix, often involving hydrogen bonding. Electrostatic forces may arise between positively or negatively charged sites on the complex and complementary charges on the DNA molecule. Gaining insight into the type and strength of these interactions is essential for evaluating the biological potential of metal-based compounds and for optimizing their structures toward selective and effective action.

**Figure 9:**
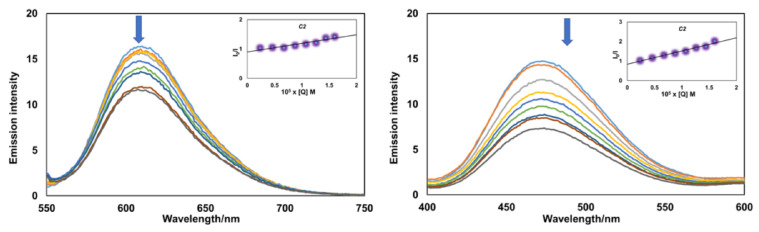
Emission spectra of EB/Hoe bound to DNA in the presence of **C2**. [EB] = 4.8 × 10^−6^ M; [Hoe] = 4.8 × 10^−6^ M; [DNA] = 4.8 × 10^−6^ M; [complex] = 0 –1.6 × 10^−5^ M; λ_ex_ = 527 nm; λ_ex_ = 346 nm. Arrows show the intensity changes upon increasing the concentration of complex. Inset graph: Plot of *I*_0_/*I* vs. [Q].

In that regard, the interaction of complexes **C1** and **C2** with DNA was investigated using fluorescence quenching assays, employing ethidium bromide (EB) as an intercalation indicator and Hoechst 33258 (Hoe) as a minor groove binding probe. Gradual addition of the increasing concentration of complexes to the EB–DNA and Hoe–DNA systems led to a decrease in fluorescence intensity (at 610 nm for EB and 470 nm for Hoe in the case of **C2**), Figure 9. The similar behavior was confirmed for the **C1** complex (Figure S1, SI). The observed fluorescence quenching suggests that the complexes are capable of interacting with DNA through both intercalative and minor groove binding modes, as evidenced by their ability to displace bonded EB and Hoe for DNA.

The strength of the interaction between **C1** and **C2** with DNA was estimated based on the Stern–Volmer constant values, presented in Table 5. The calculated *K*_sv_ values, of order magnitude 10^4^–10^5^ M^−1^, indicate a moderate to high ability of the complexes to displace EB or Hoe bounded to DNA. Furthermore, the results revealed that both complexes have a higher affinity for binding to DNA through the minor groove mode compared to intercalation. The reactivity order of the complexes is **C1** > **C2**, indicating that the complex containing copper as the metal center exhibited greater reactivity compared to the complex with palladium.

### 2.10. Comparative Molecular Docking Study of C1 and C2 Complexes at DNA Binding Sites

The docking studies of **C1** and **C2** with DNA duplexes revealed distinct binding affinities and stabilization patterns toward both 6-bp-DNA and 10-bp-DNA sequences (Table 6). The results demonstrate that both complexes interact favorably with the DNA backbone, but with differing strengths and binding modes.

For the 6-bp-DNA, compound C1 exhibited a slightly more favorable ΔG_bind_ compared to C2. The lower K_i_ and more negative ΔG_bind_ values indicate that C1 establishes more stable interactions, likely involving coordination or electrostatic attraction between the metal center and the DNA phosphate groups. The van der Waals and hydrogen-bonding terms (Δ*G*_vdw_ and Δ*G*_hbond_) contributed dominantly to the overall stabilization, while electrostatic and torsional contributions were minimal.

When docked with 10-bp-DNA, both complexes showed enhanced binding affinity relative to the shorter sequence, suggesting that longer DNA strands provide additional stabilization sites. Compound C1 again exhibited stronger binding than C2. The pronounced increase in affinity for both complexes implies that the interaction mechanism is mainly groove-binding, supported by dispersion and hydrogen-bonding effects that enhance complex stabilization along the DNA grooves.

Theoretical predictions are in good agreement with experimental fluorescence-quenching data (Table 5). The higher K_sv_ obtained for C1 in both EB–DNA (4.7 × 10^4^ M^−1^) and Hoe–DNA (1.9 × 10^5^ M^−1^) assays corroborates its superior binding capacity compared to C2, which exhibited lower K_sv_ values (2.9 × 10^4^ M^−1^ and 6.8 × 10^4^ M^−1^, respectively). This parallel between experimental and theoretical data validates the docking results. It confirms that C1 forms more stable and specific interactions with DNA, whereas C2, despite displaying similar binding behavior, shows slightly weaker stabilization. Taken together, these findings suggest that C1 possesses a higher DNA-binding propensity, capable of forming compact and energetically favorable complexes, while C2 interacts less tightly but retains the capacity to engage both short and long DNA fragments. The consistency between computational and experimental results supports the proposed groove-binding mode as the dominant interaction mechanism for both complexes.

**Figure 10:**
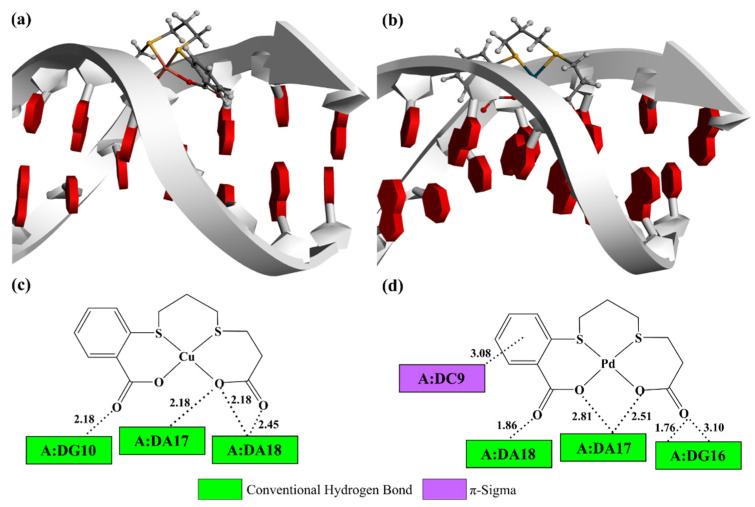
The most favorable docking poses (up) and key interactions (down) of C1 (**a**,**c**) and C2 (**b**,**d**) in the dodecamer d(CGCGAATTCGCG)2 (PDB code: 1BNA). The figure illustrates the helically twisted sugar–phosphate backbones of both complementary strands (white ribbons) and nucleobases (red). Distances between interacting nucleotides obtained from molecular docking are labeled (DA = deoxyadenosine, DG = deoxyguanosine, DC = deoxycytidine). Interaction types are color-coded (legend).

The docking analysis of **C1** and **C2** with the DNA dodecamer d(CGCGAATTCGCG)_2_ revealed distinct interaction profiles contributing to the stabilization of the resulting complexes, as illustrated in Figure 10. For compound **C1**, the most favorable binding position was predominantly groove-binding, accompanied by the formation of three key hydrogen bonds with A:DG10, A:DA17, and A:DA18. These short hydrogen bonds indicate strong polar interactions that stabilize the complex within the minor groove of DNA. The well-defined orientation of **C1** suggests that its planar structure enables efficient alignment with the DNA backbone, allowing close contact with the phosphate groups and nucleobases.

In contrast, compound C2 displayed a mixed binding mode, combining groove binding with partial intercalation between adjacent base pairs. The complex forms multiple hydrogen bonds with A:DA17, A:DA18, and A:DG16, as well as a π–sigma interaction with A:DC9. This combination of polar and π-type interactions reinforces the stability of the C2–DNA complex, while the observed intercalative contribution suggests the possibility of local DNA distortion, which could affect biological processes such as replication and transcription.

Overall, the interaction patterns reveal that both complexes exhibit strong affinity toward DNA, but with different stabilization mechanisms. C1 primarily relies on hydrogen bonding and groove interactions, resulting in a compact and energetically favorable complex, whereas C2 displays additional π-based interactions and mixed binding behavior, reflecting greater structural adaptability. These findings are consistent with the thermodynamic data (Table 3), where both compounds show negative binding free energy, confirming the spontaneity and stability of their association with DNA.

### 2.11. Albumin Binding Studies

**Figure 11:**
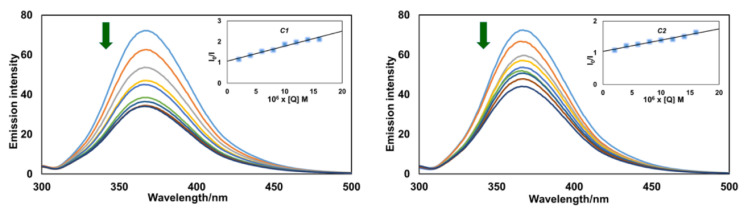
Emission spectra of BSA in the presence of **C1** and **C2**. [BSA] = 2 μM, [complex] = 0–16 μM, λ_ex_ = 295 nm. Arrows show the intensity changes upon increasing the concentrations of complex. Inset graph: Plot of *I*_0_/*I* vs. [Q].

Studying the interaction between transition metal complexes and biologically relevant molecules offers valuable insights into their potential biological mechanisms. Bovine serum albumin (BSA) is widely used as a model due to its structural similarity to human serum albumin (HSA). BSA contains two intrinsically fluorescent tryptophan residues: one exposed on the protein surface (Trp-134) and another located within a hydrophobic pocket in the second domain (Trp-212) [30]. In this study, fluorescence spectroscopy was employed to investigate the interaction of compounds C1 and C2 with BSA. Upon the addition of C1 or C2 to the BSA solution (2 μM), a notable reduction in the intrinsic fluorescence intensity of BSA at 366 nm was observed (Figure 11). The observed fluorescence quenching suggests structural rearrangements within the tertiary structure of BSA, most likely caused by changes in the local environment of tryptophan residues upon binding of the compounds to the protein [31]. The fluorescence quenching behavior of C1 and C2 was further analyzed based on the parameters such as the Stern–Volmer quenching constants (*K*_sv_), bimolecular quenching rate constants (*k*_q_), binding constants (*K*), and the number of binding sites (*n*) per BSA molecule (Table 7). These values were calculated in the manner described in the Experimental part from the plots in Figure 11 and Figure S2, SI.

The values of *K*_sv_, *k*_q_ and *K* indicate that C1 has a higher affinity toward BSA than C2, whereas the *n* values suggest that there is one site accessible for their binding. The determined *k*_q_ values for both investigated compounds are higher than the diffusion-controlled quenching rate constant (2 × 10^10^ M^−1^ s^−1^ [32,33]), suggesting that the quenching process follows a static mechanism. The binding constants (*K*) are within a favorable range. Their sufficiently high values facilitate stable interaction with albumin, supporting efficient transport through the bloodstream. Nevertheless, as the binding constants remain significantly lower than 10^15^ M^−1^—the benchmark for extremely strong non-covalent interactions—the complexes maintain the capacity to release from albumin at the site of action, thereby enabling their intended biological function [34].

### 2.12. Molecular Interaction Profiling of C1 and C2 Complexes at Multiple Binding Domains of BSA

The molecular docking results obtained for organometallic compounds C1 and C2 at the three distinct binding sites of BSA reveal clear differences in their affinities and interaction patterns, as summarized in Table 8. The examined binding regions—sites I (IIA), II (IIIA), and III (IB)—are well-known for accommodating various endogenous and exogenous ligands, making them suitable targets for evaluating the transport and binding capabilities of small bioactive molecules [35,36,37,38].

Among the tested compounds, C2 exhibited slightly stronger overall binding affinities across the analyzed domains, particularly at site I (IIA), with a binding free energy (ΔG_bind_) of −7.87 kcal/mol and an inhibition constant (K_i_) of 1.71 μM, compared to C1, which showed ΔG_bind_ = −7.68 kcal/mol and K_i_ = 2.36 μM. The most favorable binding for C1 was observed at site III (IB), with Δ*G*_bind_ = −8.45 kcal/mol and K_i_ = 0.64 μM, indicating a high affinity that could correspond to specific hydrogen bonding and van der Waals stabilization within that region. The consistently negative Δ*G*_bind_ values for both C1 and C2 indicate that all binding processes are thermodynamically spontaneous and energetically favorable. Minor differences in electrostatic contributions (ΔG_elc_) suggest that van der Waals and hydrophobic forces play dominant roles in stabilizing these complexes, typical for albumin–ligand interactions.

When these theoretical findings are compared with experimental binding parameters presented in Table 7, a strong correlation is evident. Experimentally derived binding constants (K) for BSA–ligand interactions support the computational results: C1 displayed a higher association constant (8.5 × 10^4^ M^−1^) and binding stoichiometry factor (n = 0.97) than C2 (5.3 × 10^4^ M^−1^, n = 0.83). This experimental evidence aligns with the docking outcomes, confirming that C1 forms more stable and specific complexes with albumin, particularly at the site III domain. The consistency between the theoretical and experimental data validates the docking methodology and highlights the reliability of the predicted binding modes.

**Figure 12:**
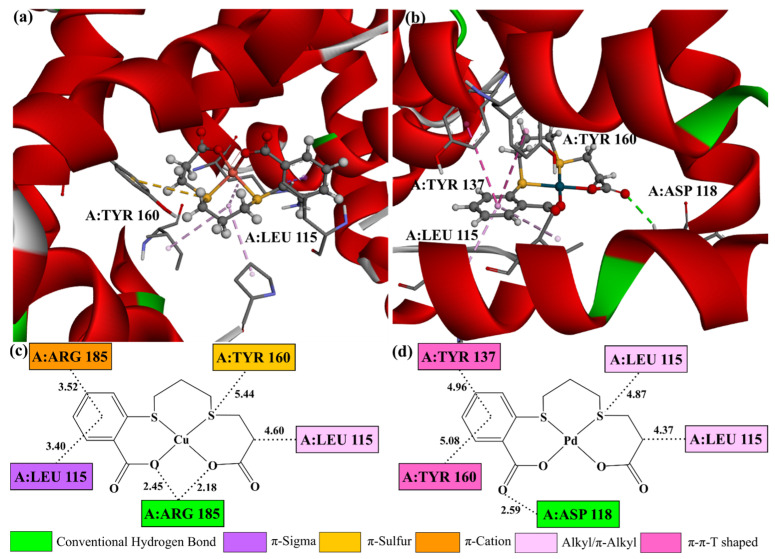
The most favorable docking poses (up) and key interactions (down) of C1 (**a**,**c**) and C2 (**b**,**d**) within the binding site III of Bovine Serum Albumin (BSA) (PDB code: 4F5S). For better clarity, the remaining part of the protein structure is excluded. Different colours represent different types of interactions (legend).

To gain deeper insight into the molecular recognition and stabilization mechanisms between the investigated organometallic complexes and BSA, the most favorable docking conformations of C1 and C2 within binding site III were analyzed (Figure 12). For compound C1, the most significant contacts include hydrogen bonds with A:ARG 185, as well as π–sulfur and π–sigma interactions with A:TYR 160 and A:LEU 115, accompanied by an additional electrostatic π–cation interaction with A:ARG 185. These interactions contribute to a well-defined ligand orientation within the binding pocket and explain the strongly negative binding energy obtained from theoretical calculations.

In contrast, compound C2 primarily forms hydrogen bonds with A:ASP 118, while π–π T-shaped and π–alkyl interactions with A:TYR 137, A:TYR 160, and A:LEU 115 stabilize its orientation within the hydrophobic cavity (Figure 12). Although the overall binding pattern of C2 resembles that of C1, the reduced contribution of hydrogen bonds and fewer polar contacts account for its lower binding affinity toward albumin. Overall, these intermolecular interactions collectively determine the thermodynamic stability and specificity of the studied complexes. The combination of polar and non-polar forces indicates that both ligands, C1 and C2, exhibit a strong tendency to bind to albumin, with C1 forming a more compact and energetically favorable complex, consistent with the experimental findings.

## 3. Materials and Methods

### 3.1. Reagents and Instruments

All reagents and solvents were obtained from commercial suppliers and used without further purification unless stated otherwise. Potassium-tetrachloridopalladate(II), 3-mercaptopropionic acid, thiosalicylic acid and copper(II)-nitrate trihydrate were obtained from Sigma-Aldrich. The elemental composition of the synthesized compounds was confirmed by elemental microanalysis. The structures of the ligand and its corresponding Cu(II) and Pd(II) complexes were characterized by infrared (IR) and nuclear magnetic resonance (NMR) spectroscopy, as well as by molar conductivity measurements.

Elemental analyses (C, H, N, and S) were performed using a Vario III CHNS Elemental Analyzer; CHS mode (Elementar Analysensysteme GmbH, Langenselbold, Germany). Infrared spectra were recorded on a Perkin-Elmer FTIR 31725-X spectrophotometer (PerkinElmer Inc., Norwalk, CT, USA) using the KBr pellet technique in the range of 4000–400 cm^−1^. ^1^H and ^13^C NMR spectra were acquired on a Varian Gemini-200 NMR spectrometer (Palo Alto, CA, USA) at 22 °C using DMSO-d_6_ as solvent and tetramethylsilane (TMS) as internal standard. The spectra were recorded from 10 mM solutions of the complexes. Molar conductivity measurements were carried out at room temperature using a Coronation digital conductivity meter with freshly prepared 10^−3^ M DMSO solutions of the complexes. Mass spectra were recorded on the Mass spectrometer Orbitrap Exploris 240 with UlitiMate 3000 RSLC nano system; MS240/250A (Thermo Fisher Scientific, Bremen, Germany) using CH_3_CN as solvent in positive mode. Ethidium bromide (EB) [3,8-diamino-5-ethyl-6-phenylphenanthridinium bromide], Hoechst 33258 (Hoe), calf thymus DNA (CT-DNA), and bovine serum albumin (BSA) were purchased from Sigma Chemical Co. and utilized as received, without any further purification. A stock solution of CT-DNA was prepared in phosphate-buffered saline (PBS, 0.01 M, pH 7.4; Sigma-Aldrich). The purity of the DNA was verified by measuring the absorbance ratio at 260 nm and 280 nm (A260/A280), which ranged from 1.8 to 1.9, indicating negligible protein contamination. DNA concentration was quantified spectrophotometrically using a molar extinction coefficient of 6600 M^−1^ cm^−1^ at 260 nm. Similarly, a BSA stock solution was prepared in 0.01 M PBS (pH 7.4), with the protein concentration adjusted to 2 μM.

The binding interactions of the complexes with calf thymus-DNA (CT-DNA) and bovine serum albumin (BSA) were examined using fluorescence spectroscopy. Fluorescence emission spectra were recorded on a Shimadzu RF-1501 PC spectrofluorometer (Shimadzu Corporation, Kyoto, Japan).

### 3.2. Syntheses of Investigation Compounds

#### 3.2.1. Preparation of the Tetradentate S,O-Ligand, Derivative of Thiosalicylic and Thiopropionic Acid (L)

A solution of sodium hydroxide (1.00 g, 25 mmol) in 40 mL of distilled water was prepared with continuous stirring. Subsequently, 25 mL of ethanol was added, followed by 1.54 g (10 mmol) of thiosalicylic acid, yielding Solution 1. Separately, a solution of 1,3-dibromopropane (10.3 g, 50 mmol, large excess) in 60 mL of ethanol was prepared (Solution 2). Solution 1 was added dropwise to Solution 2 over a period of 3–4 h under constant stirring. The reaction mixture was then stirred at room temperature overnight. After completion of the reaction, the mixture was transferred to an open beaker and evaporated with continuous stirring under air until all residual ethanol and dibromopropane had been removed (as indicated by the absence of odor). In a separate vessel, thiopropionic acid (3.19 g, 30 mmol) was dissolved in 40 mL of distilled water, followed by the addition of sodium hydroxide (2.60 g, 35 mmol), affording Solution 3. The reaction mixture obtained from Solutions 1 and 2 was then added slowly to Solution 3 over 4–5 h with continuous stirring, after which the mixture was stirred for an additional 24 h at room temperature. The resulting reaction mixture was transferred to a beaker and evaporated under a slow stream of air with constant stirring until all ethanol had been removed. The residue was diluted with 60 mL of distilled water and acidified with 2 M HCl. The resulting white, milky suspension was stirred for 10 min and then filtered through a Büchner funnel without a vacuum. The collected white solid was thoroughly washed with distilled water to remove any unreacted thiopropionic acid and dried at room temperature until a constant mass was obtained.

Yield: 2.4981 g (83.27%). Anal. Calc. for ligand (L) [TS-3-Tp] = C_13_H_16_O_4_S_2_ (Mr = 300.258): C, 51.99; H, 5.37; S, 21.31. Found: C, 52.71; H, 5.06; S, 19.93. Observed mass: 323.0382 (error: 0.15).

IR (KBr, cm^−1^): 3926, 3725, 3705, 3072, 3010, 2944, 2925, 2860, 2746, 2670, 2593, 2527, 1965, 1932, 1901, 1809, 1700, 1672, 1586, 1565, 1465, 1447, 1432, 1411, 1402, 1350, 1316, 1302, 1287, 1271, 1256, 1203, 1170, 1155, 1112, 1063, 1047, 1002, 951, 885, 871, 812, 787, 766, 740, 728, 704, 685, 662, 652, 591, 551, 519, 493, 470, 460, 454.

^1^H NMR (200 MHz, DMSO-*d*_6_, δ ppm): 1.78–1.98 (m, 2H, C9H_2_), 2.57–2.72 (m, 4H, C10H_2_, C12H_2_); 2.83–2.92 (m, 2H, C8H_2_), 2.93–3.12 (m, 2H, C11H_2_), 7.15–7.90 (m, 4H, Ar), 12.02–12.85 (s, 2H, COOH). ^13^C NMR (50 MHz, DMSO-*d*_6_, δ ppm): 26.52 C11, 28.06 C9, 29.84 C10, 30.36 C8, 34.71 C12, 125,76 C2, 126.13 C6, 128.65 C4, 131.18 C7, 132.54 C5, 140.69 C3, 167.70 C1, 175.28 C13.

#### 3.2.2. Preparation of the Copper(II) Complex with Tetradentate S,O-Ligand, Derivative of Thiosalicylic and Thiopropionic Acid (C1)

The copper(II) complex with the tetradentate S,O-ligand derived from thiosalicylic and thiopropionic acids (C1) was synthesized by direct reaction of Cu(NO_3_)_2·_3H_2_O, the corresponding ligand (L), and lithium hydroxide in a molar ratio of 1:1:2. Cu(NO_3_)_2_·3H_2_O (0.1000 g, 0.4139 mmol) was dissolved in 10.00 mL of distilled water under heating on a steam bath. Subsequently, the tetradentate S,O-ligand (L) (0.1243 g, 0.4139 mmol) was added to the reaction mixture with continuous stirring. The mixture was maintained under stirring and heating for 3 h, during which time an aqueous solution of lithium hydroxide (0.0192 g, 0.8278 mmol in distilled water) was added gradually in small portions. Upon completion of the reaction, a blue-green precipitate corresponding to the copper(II) complex (C1) was formed. The solid was filtered, thoroughly washed with distilled water to remove residual reagents, followed by successive washing with ethanol and diethyl ether to eliminate any remaining organic impurities or side products, and finally dried under vacuum to constant mass.

Yield: 0.1186 g (79.18%). Anal. Calc. for (C1) [Cu(TS-3-Tp)] = CuC_13_H_14_O_4_S_2_ (Mr = 361.92): C, 43.14; H, 3.90; S, 17.72. Found: C, 43.50; H, 4.21; S, 17.64. Observed mass: 362.9785 (error: 0.00). Molar conductivity 10.10 S·cm^−2^·mol^−1^.

IR (KBr, cm^−1^): 3926, 3422, 3055, 2921, 2857, 2648, 2235, 1698, 1614,1588,1462,1433, 1402, 1313, 1282, 1255, 1154, 1062, 1046, 1004, 954, 848, 798, 748, 720. 696, 659, 613, 509, 501.

#### 3.2.3. Preparation of the Palladium(II) Complex with Tetradentate S,O-Ligand, Derivative of Thiosalicylic and Thiopropionic Acid (C2)

The palladium(II) complex with the tetradentate S,O-ligand derived from thiosalicylic and thiopropionic acids (C2) was synthesized by direct reaction of potassium tetrachloridopalladate(II), the corresponding ligand (L), and lithium hydroxide in a molar ratio of 1:1:2. K_2_[PdCl_4_] (0.1000 g, 0.3065 mmol) was dissolved in 10.00 mL of distilled water under heating on a steam bath. Subsequently, the tetradentate S,O-ligand (L) (0.0921 g, 0.3065 mmol) was added to the reaction mixture, which was stirred continuously. The mixture was maintained under stirring and heating for 3 h, during which time an aqueous solution of lithium hydroxide (0.0256 g, 0.6130 mmol in distilled water) was added gradually in small portions. After completion of the reaction, a yellow precipitate corresponding to the palladium(II) complex (C2) was formed. The solid was filtered, thoroughly washed with distilled water to remove residual reagents, followed by successive washing with ethanol and diethyl ether to eliminate any remaining organic impurities or side products, and finally dried under vacuum to constant mass.

Yield: 0.0740 g (59.61%). Anal. Calc. for [Pd(TS-3-Tp)] = PdC_13_H_14_O_4_S_2_ (Mr = 404.79): C, 38.57; H, 3.49; S, 15.84. Found: C, 38.99; H, 3.80; S, 16.01. Observed mass: 404.9446 (error: 1.16).

Molar conductivity 8.59 S cm^−2^ mol^−1^.

IR (KBr, cm^−1^): 3931, 3725, 3682, 3441, 3096, 2994, 2979, 2932, 2920, 2625, 1947, 1720, 1581, 1553, 1458, 1419, 1403, 1349, 1256, 1235, 1178, 1159, 1147, 1117, 1039, 919, 897, 868, 838, 799, 745, 719, 682, 668, 641, 629, 592, 550, 518, 503, 483.

^1^H NMR (200 MHz, DMSO-*d*_6_, δ ppm): 1.78–1.95 (m, 2H, C9H_2_), 2.60–3.01 (m, 4H, C10H_2_, C12H_2_); 3.03–3.12 (m, 2H, C8H_2_), 3.47–3.68 (m, 2H, C11H_2_), 7.15–7.92 (m, 4H, Ar). ^13^C NMR (50 MHz, DMSO-*d*_6_, δ ppm): 26.91 C11, 29.95 C9, 29.77 C10, 30.49 C8, 34.62 C12, 124,17 C2, 125.75 C6, 127.65 C4, 131.14 C7, 132.47 C5, 140.32 C3, 167.28 C1, 173.27 C13.

### 3.3. Cytotoxic Activity

#### 3.3.1. Cell Culture

For investigation of cytotoxic effects of C1, C2 and L, we employed a variety of cell lines, including mouse triple-negative breast carcinoma (4T1), human breast carcinoma (MCF-7), mouse colorectal carcinoma (CT26), human colorectal carcinoma (HCT116), and mouse mesenchymal stem cells (mMSCs), all obtained from the American Type Culture Collection (ATCC, USA). All cells were maintained in complete high-glucose Dulbecco’s Modified Eagle Medium (DMEM, Sigma Aldrich, Munich, Germany) containing 10% fetal bovine serum (FBS, Sigma Aldrich), penicillin G (100 IU/mL, Sigma Aldrich), and streptomycin (100 μg/mL, Sigma Aldrich) in the atmosphere with 5% CO_2_ at 37 °C at absolute humidity. The assessment of cell number and viability was conducted using trypan blue staining. The cell suspensions with 95% viable cells were used for the experiment.

#### 3.3.2. Assessment of C1, C2 and L Cytotoxicity

The tumoricidal activities of C1, C2, and L were analyzed by MTT (3-(4,5-dimethylthiazol-2-yl)-2,5-diphenyl tetrazolium bromide) assay and compared with the cytotoxicity of cisplatin as the conventional chemotherapeutic, following previously established protocol [39]. Tumor cells (5 × 10^3^ cells/well) were seeded into 96-well cell culture plates and allowed to adhere. After 24 h, the cells were exposed to two-fold growing concentrations of C1, C2, and L or cisplatin (ranging from 3.9 to 500 μM) for 48 h at 37 °C in the atmosphere containing 5% CO_2_ at absolute humidity. Control wells contained complete cell culture medium alone. Further, the medium was removed and MTT solution in PBS was added to each well and incubated for an additional 2 h at 37 °C in 5% CO_2_. The cell-free supernatants were suctioned off, and DMSO (150 μL) and glycine buffer (20 μL) were added to dissolve the formazan crystals. The plates were shaken for 10 min. The optical density was determined at 595 nm using a Zenyth 3100 multimode microplate detector. For IC_50_ determination, each concentration was evaluated in technical triplicate within each experiment, and the experiments were repeated independently three times (biological replicates). IC_50_ values were calculated using nonlinear regression analysis of dose–response curves and expressed as mean ± standard deviation (SD). Additional independent replicate experiments were performed for 4T1 and CT26 cells to further evaluate reproducibility of the C1 IC_50_ values. The IC_50_ values (representing the concentration required to reduce cell viability by 50%), were calculated using Microsoft Excel 2010. The percentage of cytotoxicity was calculated as previously described [40].

#### 3.3.3. Detection of Apoptosis

Apoptosis in treated 4T1 cells was assessed using a dual staining assay with Annexin V and Propidium Iodide (PI) following previously established protocols [41]. 4T1 cells were cultured under standard conditions until approximately 80% confluence was reached. For apoptosis detection, 4T1 were exposed to the IC50 dose of C1. After 24h of the treatment with tested compound, the cells were harvested, washed two times in ice cold PBS, and double stained with fluorescein isothiocyanate (FITC) labeled Annexin V (BD Pharmingen, CA, USA) and Propidium Iodide (PI) (Sigma-Aldrich) in ice cold binding buffer (10× binding buffer: 0.1 M Hepes/NaOH (pH 7.4), 1.4 M NaCl, 25 mM CaCl_2_) at the room temperature in the dark for 15 min. Apoptotic and necrotic cell populations were then analyzed using flow cytometry, which distinguishes cell types based on fluorescence profiles. Flow cytometric analysis generated dot plots illustrating fluorescence distribution, enabling the calculation of cell populations in each quadrant. This approach classified cells into four categories: live cells (Annexin V^−^/PI^−^), early apoptotic cells (Annexin V^+^/PI^−^), late apoptotic cells (Annexin V^+^/PI^+^), and necrotic cells (Annexin V^−^/PI^+^).

In subsequent experiments, the 4T1 cells were fixed and permeabilized using a permeabilization buffer (BD Bioscience, Heidelberg, Germany). They were then incubated with antibodies specific to Bcl-2, Bax, and caspase-3 (Thermo Fisher Scientific Inc., Waltham, MA, USA), following protocols outlined in prior research [41]. The treated cells were analyzed using a FACS Calibur flow cytometer (BD Biosciences, San Jose, CA, USA), and the acquired data were thoroughly processed using FlowJo software (version 10.7.2, Tree Star, Inc., Ashland, OR, USA).

#### 3.3.4. Analysis of Mitochondrial Membrane Potential Using JC-10 Immunofluorescence Staining

The JC-10 dye was used to evaluate mitochondrial membrane potential. 4T1 cells were plated in 24-well plates at a density of 2 × 10^4^ cells per well and incubated for 24 h at 37 °C with 5% CO_2_. Cells were treated for 48 h with C1 and CDDP at their respective IC_50_ values. The cells were stained with 2.5 μM JC-10 dye for 20 min in warm PBS at 37 °C and 5% CO_2_, then rinsed with warm 1× PBS. The cellular localization and fluorescence of JC-10 in the cells were observed using a Gramma Libero FLUO500T trinocular inverted fluorescence microscope. For quantification, we used the ratio of fluorescence emissions at 525 and 590 nm. We used the 2020 S-EYE Setup Microscope camera and S-EYE_Setup-1.6.0.11 software to capture images. The images were examined using ImageJ software.

#### 3.3.5. Immunofluorescence Analysis of Cytochrome c

4T1 cells were seeded at a density of 1 × 10^4^ cells per well in 24-well cell culture plates and treated with C1 and cisplatin (CDDP) at their respective IC_50_ concentrations for 48 h. Cells were then fixed for 30 min at room temperature in 4% paraformaldehyde, permeabilized for 30 min in 0.2% Tween 20 in PBS, and blocked for 30 min in 0.1% Tween 20 in PBS. The cells were incubated with a monoclonal cytochrome c antibody (37BA11, Invitrogen (Waltham, MA, USA), 1:100) for one hour at room temperature. Following this, the cells were treated with the secondary antibody, Alexa Fluor 488 (Invitrogen, #A32766, 1:200), and counterstained with propidium iodide (PI). An inverted FLUO500T trinocular fluorescent microscope (Gramma Libero, Belgrade, Serbia) was used to evaluate the localization of cytochrome c within the cells. We analyzed the images using ImageJ software, setting the scale to 100 μm and applying a magnification of 40×.

#### 3.3.6. Detection of Acidic Autophagic Vacuoles

4T1 cells were treated with the IC_50_ dose of C1 and CDDP for 48 h. After treatment, the cells were stained with 2 μg/mL acridine orange (AO) for 15 min. The cells were then analyzed for the presence of autophagic vacuoles, which appeared orange-red, using a FLUO500T trinocular inverted fluorescent microscope. In addition, the ImageJ program was employed to analyze the images (scale image 100 µm; 40×) that were captured by the 2020 S-EYE Setup Microscope camera using S-EYE_Setup-1.6.0.11 software. Quantification analysis was conducted using fluorescence emissions (MFI) at 525 nm and 590 nm. To evaluate the induction of autophagy by C1 and CDDP (IC_50_), cells were trypsinized, centrifuged, and subsequently stained with 1 μg/mL Acridine Orange for 30 min at room temperature. Flow cytometry analysis was conducted using a Cytomics FC500 (Beckman Coulter, Brea, CA, USA). The data was processed using FlowJo software and is presented as bar histograms.

#### 3.3.7. Analysis of the Expression of p62, and LC3B

The 4T1 cells were placed into 24-well plates with 2 × 10^4^ cells per well in a full DMEM medium. The plates were then maintained at 37 °C with 5% CO_2_ for 24 h. Subsequently, cells were treated with C1 and CDDP (IC_50_-μM) for a duration of 48 h. The cells were collected and fixed in 4% paraformaldehyde at room temperature for 30 min. Subsequently, they were permeabilized with 0.2% Tween 20 in PBS and then blocked for 30 min with 0.1% Tween 20 in PBS (blocking buffer). Then, the cells were incubated with selected primary antibodies (1:100): Anti-p62/SQSTM1 Rabbit Monoclonal Antibody (Biocompare, # M00300) and Rabbit Anti-LC3B Polyclonal Antibody (Novus Biologicals, # NB100-2220). The cells were then stained with secondary antibodies (1:200): anti-rabbit Alexa Fluor^®^ 594 Conjugate (Cell Signaling, #8889) and anti-rabbit Alexa Fluor 488 (Cell Signaling, #4412) (1:200). The labeled cells were afterwards analyzed with a Cytomics FC500 flow cytometer (Beckman Coulter, USA).

#### 3.3.8. The Analysis of Cell Cycle Profile

Flow cytometry was used to investigate the effects of C1 and CDDP (IC_50_-µM) on the cell cycle in 4T1 cells after a 48 h incubation. Untreated 4T1 cells, as well as those treated with C1 and CDDP, were collected, centrifuged, and rinsed with PBS. Subsequently, cold ethanol was slowly added to the cells to achieve a final concentration of 70%. Cells were kept on ice for at least 4 h, then washed with PBS and mixed in a staining solution (PBS with 100 μg/mL RNaseA and 50 μg/mL Propidium Iodide-PI) in the dark at room temperature for 15–30 min. Flow cytometry was employed to evaluate the cell cycle characteristics of the cells. The percentages of cells allocated to the G0/G1, S, and G2/M phases were calculated by the FlowJo software and displayed by histogram bars.

#### 3.3.9. The Analysis of the Cell Cycle Regulators

In this part of the study, we delved into the impact of the C1 complex on proliferation potential and cell cycle regulators in 4T1 cancer cells, following protocols established in previous studies [31]. Cells were treated with C1 complex at its IC50 concentration for 24 h, while a control group was cultured without treatment. The treated cells were fixed and permeabilized with permeabilization buffer (BD Bioscience) and incubated with specific antibodies, including Ki-67 (11-5698-82, eBioscience), anti-mouse cyclin D antibody (#MA5-28534, Thermo Fisher Scientific) and allophycocyanin (APC) conjugated anti-mouse protein kinase AKT phosphorylated on S473 (pAKT) antibody (#17-9715-42, eBioscience). Flow cytometry was performed on a FACSCalibur flow cytometer (BD Biosciences). The data were analyzed using FlowJo software (Tree Star).

#### 3.3.10. Evaluation of Migratory Capacity

The migratory capacity of tumor cells in vitro was evaluated using the wound scratch assay in accordance with previously established protocols [42]. The 4T1 cell line was cultured in three six-well plates until a confluent monolayer was formed. A linear scratch was created in each well using a sterile 200 μL pipette tip held at a 30-degree angle. Following scratch formation, the cells were washed with PBS, and the wells were treated with medium containing either the tested C1 complex or cisplatin. An initial image of the scratched area was taken immediately after scratch formation, followed by subsequent images of the same area captured at 0, 12, and 24 h post-treatment to monitor cell migration. Imaging was performed using an inverted optical microscope (Olympus, Tokyo, Japan) to document the scratch closure. Image analysis was conducted using ImageJ software (National Institute of Health, Bethesda, MD, USA) to measure the scratch area and calculate the percentage of wound closure [42].

#### 3.3.11. Statistical Analysis

Statistical analyses were conducted following the assessment of data distribution normality. Depending on the distribution, either a two-tailed Student’s *t*-test or the nonparametric Mann–Whitney Rank Sum test was employed, as deemed appropriate. All analyses were performed using SPSS software version 26.0 for Windows (SPSS Inc., Chicago, IL, USA). Data were reported as mean ± standard deviation (SD) or mean ± standard error (SE). A *p*-value of less than 0.05 (*p* < 0.05) was considered statistically significant.

### 3.4. Fluorescence Quenching Measurements

#### 3.4.1. DNA Binding Studies

To explore the binding affinities of the complexes toward calf thymus–DNA (CT–DNA), fluorescence spectra of EB–CT–DNA and Hoe–CT–DNA systems at a fixed concentration (4.8 × 10^−6^ M) with incremental addition of C1 and C2 (0–1.6 × 10^−5^ M) were recorded. For the EB system, fluorescence emission spectra were recorded from 550 to 750 nm following excitation at 527 nm. In the case of Hoe, emission was monitored between 400 and 600 nm, using an excitation wavelength of 346 nm. All experimental solutions were prepared in phosphate-buffered saline (PBS, pH 7.4). The fluorescence quenching behavior was analyzed using the Stern–Volmer Equation (1) [43]. In this context, I_0_ denotes the fluorescence intensity in the absence of the quencher, while I corresponds to the intensity in the presence of the quenching agents (C1 and C2). The variable [Q] represents the concentration of the quencher. The Stern–Volmer quenching constant (K_sv_) was determined from the linear slope of the plot of I_0_/I versus [Q].

I_0_/I =1 + K_sv_[Q] (1)

#### 3.4.2. Albumin-Binding Studies

Protein binding interactions were evaluated through tryptophan fluorescence quenching assays using bovine serum albumin (BSA) as the model protein. The BSA concentration was fixed at 2 µM, while test compounds C1 and C2 were introduced at varying concentrations ranging from 2 to 16 µM. This setup established a molar ratio (r = [BSA]/[compound]) between 1 and 8. Fluorescence emission spectra were collected in the wavelength range of 300–500 nm, with excitation at 295 nm. The Stern–Volmer Equation (2) [1] was utilized to calculate the quenching constant (K_sv_), which provides insight into the interaction between the quencher and BSA:

I_0_/I = 1 + k_q_τ_0_[Q] = 1 + K_sv_[Q] (2)

Here, I_0_ and I represent the fluorescence intensities of BSA in the absence and presence of the quencher, respectively; τ_0_ denotes the average lifetime of BSA fluorescence in its unbound state, approximately 10^−8^ seconds. The variable [Q] corresponds to the concentration of the quenching agent. Further, association binding constant (K) and the number of binding sites per albumin (n), were obtained using Equation (3) [44]:

(ΔI/I_0_)/[Q] = nK − KΔI/I_0_ (3)

### 3.5. Molecular Docking Study

To examine the binding affinity of the organometallic compounds C1 and C2 toward bovine serum albumin (BSA), their interaction with DNA molecules, and their inhibitory potential against the pro-apoptotic enzymes caspase-3 (CASP3) and Bcl-2-associated X protein (BAX), as well as the anti-apoptotic enzyme B-cell lymphoma 2 (BCL2), a detailed molecular docking study was conducted using AutoDock Tools 1.5.7 integrated with AutoDock 4.2.6 [45]. The molecular docking workflow was systematically designed to explore the binding preferences and inhibitory potential of the examined complexes. The structural optimization of C1 and C2 was carried out using the Gaussian16 software package [46], employing the B3LYP–D3BJ functional [47] with the 6-311+G(d,p) basis set for C, O, S, and H atoms, while metal was replaced by a generic metal center defined through the def2-TZVPD basis set [48], which includes an effective core potential, to ensure accurate geometry refinement and reliable description of electronic properties. The three-dimensional crystallographic structures of the biological targets, including BSA (PDB ID: 4F5S) [49], DNA hexamer d(CGATCG)_2_ with an intercalative binding site (PDB ID: 1Z3F) [50], DNA dodecamer d(CGCGAATTCGCG)_2_ (PDB ID: 1BNA) [51], and the enzymes CASP3 (PDB ID: 3KJF) [52], BAX (PDB ID: 2YXJ) [53], and BCL2 (PDB ID: 2W3L) [54], were obtained from the RCSB Protein Data Bank (accessed on 28 March 2024). All protein and DNA structures were prepared using BIOVIA Discovery Studio 2021 by removing non-essential heteroatoms, water molecules, and co-crystallized ligands that could interfere with ligand binding. For BSA, the grid dimensions were set to 60 × 60 × 60 Å with a grid spacing of 0.375 Å, and three binding domains were defined: site I (IIA) at −4.80 × 30.50 × 101.01 Å, site II (IIIA) at 10.91 × 16.30 × 119.72 Å, and site III (IB) at 19.86 × 33.53 × 97.92 Å. For DNA docking, the grid boxes were defined as 50 × 50 × 50 Å with a spacing of 0.375 Å for the 6-bp-DNA structure (centered at 0.871 × 17.488 × 46.277 Å) and 60 × 74 × 120 Å (grid spacing 0.375 Å) for the 10-bp-DNA structure (centered at 15.81 × 21.31 × 9.88 Å). For enzyme docking, the grid boxes were configured as follows: CASP3—64 × 64 × 64 Å^3^ (centered at 21.578 × −5.202 × −10.866 Å), BAX—66 × 66 × 66 Å^3^ (centered at 23.435 × −17.339 × 37.847 Å), and BCL2—66 × 66 × 66 Å^3^ (centered at 39.806 × 26.936 × −12.415 Å), all with a spacing of 0.375 Å. The docking simulations were performed using the Lamarckian Genetic Algorithm (LGA) [55], which efficiently combines local and global search methods and is well suited for ligand–protein docking studies involving flexible ligands and rigid receptor structures. The algorithm parameters were adjusted to ensure precision and reproducibility: a population size of 150 individuals, 2,500,000 energy evaluations, 27,000 generations, a mutation rate of 0.02, and a crossover rate of 0.8. Each docking experiment was repeated in 100 independent runs to thoroughly explore all possible binding modes and identify the most stable conformations of the examined complexes.

## 4. Conclusions

In this study, a new tetradentate S,O-donor ligand (L) derived from thiosalicylic and thiopropionic acids was synthesized and used to prepare two transition-metal complexes: a copper(II) complex (C1) and a palladium(II) complex (C2). Elemental microanalysis, IR and NMR spectroscopy, and molar conductivity measurements consistently confirmed the proposed composition and coordination mode of the metal–ligand systems, indicating the formation of electrically neutral complexes in solution. Diagnostic shifts in the carboxylate stretching vibrations in the IR spectra, together with changes in the C–S region, confirmed deprotonation and coordination through O/S donor atoms, while NMR data further demonstrated that the carboxyl groups are protonated in the free ligand and become deprotonated upon complexation.

Biological screening showed all tested compounds had dose-dependent antiproliferative effects. There were notable differences in potency and selectivity across cancer cell lines. C1 had the highest activity within tested compound series, especially against murine 4T1 triple-negative breast cancer and CT26 colorectal cancer cells. It showed lower toxicity toward non-tumor mMSCs, as reflected in the highest selectivity indices. In contrast, C2 and the free ligand L were more active against MCF-7 cells. This highlights that the metal center and coordination environment greatly influence cell-type-specific sensitivity. Therefore, the subsequent investigation focused on the detailed mechanism of C1. C1 induced apoptosis with a clear predominance of apoptotic (rather than necrotic) cell death, accompanied by mitochondrial membrane depolarization (loss of ΔΨM) and enhanced cytochrome c release, indicating activation of the intrinsic mitochondrial pathway. This was supported by a pronounced pro-apoptotic shift in the Bax/Bcl-2 ratio and a tendency toward caspase-3 activation. In addition to apoptosis, C1 strongly induced autophagy, as evidenced by acridine-orange-positive acidic vesicles and decreased levels of p62 and LC3B, suggesting altered autophagic flux in treated cells. C1 also induced G0/G1 cell-cycle arrest and suppressed key proliferative regulators, including Ki-67, cyclin D (and G1/S transition control), and phosphorylated AKT, linking the observed antiproliferative effect to inhibition of growth and survival signaling pathways. Functionally, these molecular changes resulted in a significant reduction in the migratory capacity of 4T1 cells in the wound-healing assay, indicating an anti-invasive phenotype in vitro. The results of fluorescence quenching experiments with CT-DNA demonstrated moderate to strong binding of both complexes to DNA, with a preference for minor groove interactions over intercalation and a higher binding affinity of C1 than C2. Albumin-binding experiments showed efficient quenching of BSA tryptophan fluorescence for both complexes, with kq values exceeding the diffusion-controlled limit, consistent with a predominantly static quenching mechanism and formation of stable complexes.

To elucidate the molecular basis of activity, biomolecular interaction studies were conducted, supported by molecular docking. Docking confirmed favorable DNA groove-binding conformations and stable BSA binding. This integrated chemical–biological–computational study identifies the copper(II) complex C1 as the most biologically active candidate among the compound systems investigated. However, its cytotoxic potency remained lower than that of CDDP, and the observed selectivity should be interpreted as moderate. The mechanistic experiments indicate that C1 can modulate several apoptosis-, autophagy-, and cell-cycle-related readouts in vitro. C1 also exhibits a favorable biomolecular interaction profile with DNA and serum albumin, relevant to activity and transport. Taken together, these findings identify C1 as a biologically active copper(II) complex of mechanistic interest within this compound series, providing a basis for further structural optimization and preclinical investigation.

## Figures and Tables

**Table 1:** The most important infrared bands (cm^−1^) of the investigated compounds.

Compound	-S-R	-COO^−^ (as)
S,O-tetradentate ligand, L	740 (m)	1672
Cu(II)-complex, C1	746 (m)	1614 (s), 1588 (s)
Pd(II)-complex, C2	745 (m)	1581 (s), 1553 (s)

**Table 2:** IC_50_ (μM) of tested compounds and cisplatin-treated 4T1, MCF-7, CT26, HCT116 cells and mMSCs, as determined by the MTT assay.

IC_50_ (µM) 48 h				
Cell Line/Complex	C1	C2	L	CDDP
mMSCs	487.85 ± 58.11	444.96 ± 95.33	N/A	8.33 ± 1.23
4T1	146.22 ± 28.23	388.66 ± 37.1	400 ± 21.38	12.1 ± 0.1
MCF-7	212.65 ± 49.83	60.34 ± 2.24	125.21 ± 14.12	35.16 ± 18.64
CT26	161.48 ± 24.91	293.28 ± 32.31	507.89 ± 92.56	16.44 ± 0.18
HCT116	˃500	N/A	N/A	24.32 ± 4.65

The data are presented as mean values ± SD (standard deviation) from three independent biological experiments, with each concentration tested in technical triplicate.

**Table 3:** Selectivity index (SI) of tested compounds and cisplatin-treated 4T1, MCF-7, CT26, HCT116 cells, and mMSCs.

Selectivity Index (SI) IC_50_ mMSCs/IC_50_				
Complex/Cell Line	4T1	MCF-7	CT26	HCT116
C1	3.3	2.3	3	N/A
C2	1.1	7.4	1.5	N/A
L	N/A	N/A	N/A	N/A
CDDP	0.7	0.2	0.5	0.3

**Table 4:** Thermodynamic and energetic parameters of **C1**, the active site CASP3, BCL2, and BAX, derived from molecular docking simulations including inhibition constants (*K*_i_, µM) and binding free energy (Δ*G*_bind_, kcal/mol) values calculated from different energy components: total internal energy (Δ*G*_total_), torsional free energy (Δ*G*_tor_), unbound system’s energy (Δ*G*_unb_), electrostatic energy (Δ*G*_elc_), and the sum of dispersion and repulsion (Δ*G*_vdw_), hydrogen bond (Δ*G*_hbond_), and desolvation (Δ*G*_desolv_).

Comp.	ΔG*_bind_*	K_i_ (µM)	ΔG*_inter_*	ΔG*_vdw+hbond+desolv_*	ΔG*_elec_*	ΔG*_total_*	ΔG*_tor_*	ΔG*_unb_*
C1								
CASP3	−7.17	5.53	−7.17	−7.11	0.00	0.00	0.00	0.00
BCL2	−5.88	49.25	−5.88	−5.64	−0.23	0.00	0.00	0.00
BAX	−6.01	39.36	−6.01	−5.99	−0.02	0.00	0.00	0.00

**Table 5:** Stern−Volmer constants (*K*_sv_) obtained from EB–DNA or Hoe–DNA fluorescence competition experiments.

Complex	*K*_sv_ EB (M^−1^)	*K*_sv_ Hoe (M^−1^)
C1	(4.7 ± 0.1) × 10^4^	(1.9 ± 0.1) × 10^5^
C2	(2.9 ± 0.1) × 10^4^	(6.8 ± 0.1) × 10^4^

**Table 6:** Thermodynamic and energetic parameters of C1 and C2 in hexanucleotide d(CGATCG)_2_ (6-bp–DNA) and DNA dodecamer d(CGCGAATTCGCG)_2_ (10-bp-DNA), derived from molecular docking simulations including inhibition constants (*K*_i_, µM) and binding free energy (Δ*G*_bind_, kcal/mol) values calculated from different energy components: total internal energy (Δ*G*_total_), torsional free energy (Δ*G*_tor_), unbound system’s energy (Δ*G*_unb_), electrostatic energy (Δ*G*_elc_), and the sum of dispersion and repulsion (Δ*G*_vdw_), hydrogen bond (Δ*G*_hbond_), and desolvation (Δ*G*_desolv_).

Comp.	ΔG*_bind_*	K_i_ (µM)	ΔG*_inter_*	ΔG*_vdw+_* *_hbond+_* *_desolv_*	ΔG*_elec_*	ΔG*_total_*	ΔG*_tor_*	ΔG*_unb_*
6-bp-DNA								
C1	−6.98	7.62	−6.98	−6.87	−0.11	0.00	0.00	0.00
C2	−6.82	10.06	−6.82	−6.80	−0.02	0.00	0.00	0.00
10-bp-DNA								
C1	−7.98	1.57	−7.92	−7.70	−0.22	0.00	0.00	0.00
C2	−7.83	1.83	−7.83	−7.62	−0.20	0.00	0.00	0.00

**Table 7:** The BSA binding constants and parameters (*K*_sv_, *k*_q_, *K*, *n*) derived for **C1** and **C2**.

Comp.	*K*_sv_ (M^−1^)	*k*_q_ (M^−1^ s^−1^)	*K* (M^−1^)	*n*
C1	(7.2 ± 0.1) × 10^4^	(7.2 ± 0.1) × 10^12^	(8.5 ± 0.1) × 10^4^	0.97
C2	(3.5 ± 0.1) × 10^4^	(3.5 ± 0.1) × 10^12^	(5.3 ± 0.1) × 10^4^	0.83

**Table 8:** Thermodynamic and energetic parameters of C1 and C2 to three distinct active sites of Bovine Serum Albumin (BSA), derived from molecular docking simulations including inhibition constants (*K*_i_, µM) and binding free energy (Δ*G*_bind_, kcal/mol) values calculated from different energy components: total internal energy (Δ*G*_total_), torsional free energy (Δ*G*_tor_), unbound system’s energy (Δ*G*_unb_), electrostatic energy (Δ*G*_elc_), and the sum of dispersion and repulsion (Δ*G*_vdw_), hydrogen bond (Δ*G*_hbond_), and desolvation (Δ*G*_desolv_).

Conformations	ΔG*_bind_*	K_i_ (µM)	ΔG*_inter_*	ΔG*_vdw+hbond+desolv_*	ΔG*_elc_*	ΔG*_total_*	ΔG*_tor_*	ΔG*_unb_*
C1								
BSA-I	−7.68	2.36	−7.68	−7.67	0.00	0.00	0.00	0.00
BSA-II	−7.39	3.82	−7.39	−7.35	−0.04	0.00	0.00	0.00
BSA-III	−8.45	0.64	−8.45	−8.43	−0.02	0.00	0.00	0.00
C2								
BSA-I	−7.87	1.71	−7.87	−7.81	−0.05	0.00	0.00	0.00
BSA-II	−6.97	7.75	−6.97	−6.57	−0.40	0.00	0.00	0.00
BSA-III	−7.71	2.25	−7.71	−7.69	−0.01	0.00	0.00	0.00

## Data Availability

The data are available upon request.

## Supplementary Materials

The following supporting information can be downloaded at: https://www.mdpi.com/article/10.3390/ijms27135659/s1.

## Abbreviations

The following abbreviations are used in this manuscript:

4T1	Mouse triple-negative breast carcinoma cell line
AKT	Protein kinase B
AnnV	Annexin V
AO	Acridine Orange
Ar	Aromatic ring
BAX	BCL2-associated X protein
BCL2	B-cell lymphoma 2
BSA	Bovine serum albumin
CASP3	Caspase-3
CDDP	Cisplatin
CDK2	Cyclin-dependent kinase 2
CDK4	Cyclin-dependent kinase 4
CDK6	Cyclin-dependent kinase 6
CT26	Mouse colorectal carcinoma cell line
CT-DNA	Calf thymus deoxyribonucleic acid
Cu(II)	Copper(II)
ΔG_bind	Binding free energy
ΔG_desolv	Desolvation free energy
ΔG_elec	Electrostatic free energy
ΔG_hbond	Hydrogen bond contribution to binding free energy
ΔG_inter	Interaction free energy
ΔG_tor	Torsional free energy
ΔG_total	Total free energy
ΔG_unb	Unbound system free energy
ΔG_vdw	van der Waals free energy
ΔΨM	Mitochondrial membrane potential
DG	Deoxyguanosine
DA	Deoxyadenosine
DC	Deoxycytidine
DT	Deoxythymidine
DMSO	Dimethyl sulfoxide
DNA	Deoxyribonucleic acid
EB	Ethidium bromide
FACS	Fluorescence-activated cell sorting
FITC	Fluorescein isothiocyanate
FTIR	Fourier-transform infrared spectroscopy
G0/G1	Gap 0/Gap 1 phase of the cell cycle
HCT116	Human colorectal carcinoma cell line
Hoe	Hoechst 33258
HSA	Human serum albumin
IC_50_	Half-maximal inhibitory concentration
IR	Infrared spectroscopy
JC-10	Mitochondrial membrane potential fluorescent probe
K	Binding constant
Ki	Inhibition constant
Ksv	Stern–Volmer quenching constant
kq	Bimolecular quenching rate constant
LC3B	Microtubule-associated protein 1A/1B-light chain 3B
MCF-7	Human breast carcinoma cell line
MFI	Mean fluorescence intensity
mMSCs	Mouse mesenchymal stem cells
mTOR	Mammalian target of rapamycin
MTT	3-(4,5-dimethylthiazol-2-yl)-2,5-diphenyltetrazolium bromide
NMR	Nuclear magnetic resonance
PBS	Phosphate-buffered saline
PI	Propidium iodide
PI3K	Phosphoinositide 3-kinase
p-AKT	Phosphorylated protein kinase B
ROS	Reactive oxygen species
SEM	Standard error of the mean
SI	Selectivity index
Trp	Tryptophan
