# Supplementary material for: Biological Activity of Copper(II) and Palladium(II) Complexes with a Tetradentate S,O-Donor Ligand
Source: Int J Mol Sci. 2026 Jun 23;27(13):5659. doi: 10.3390/ijms27135659 (PMC13360782; doi:10.3390/ijms27135659)

Supplementary Figure S1

Izvestaj Orbitrap Exploris 240 snimanja prema zahtevu D_2026_018_JPKG

Izvestaj br. R_2026_018_JPKG

Maseni spektrometar: OrbitrapExploris 240

Analizator: Orbitrap

Uzorak je uveden direktnom injekcijom.

Tip jonizacije: Heated ESI (HESI)

Polaritet: Pozitivan

Uslovi snimanja:

- Spray Voltage (V): 3600
- Vaporizer temp (°C): 50
- ITC temp (°C): 295
- Sheath Gas Flow Rate: 4
- Aux Gas Flow Rate: 1
- Sweep Gas Flow Rate: 0
- RF Lens (%): 70
- Orbitrap Resolution (at m/z 200): 45 000

OE1864 TS-3-TP

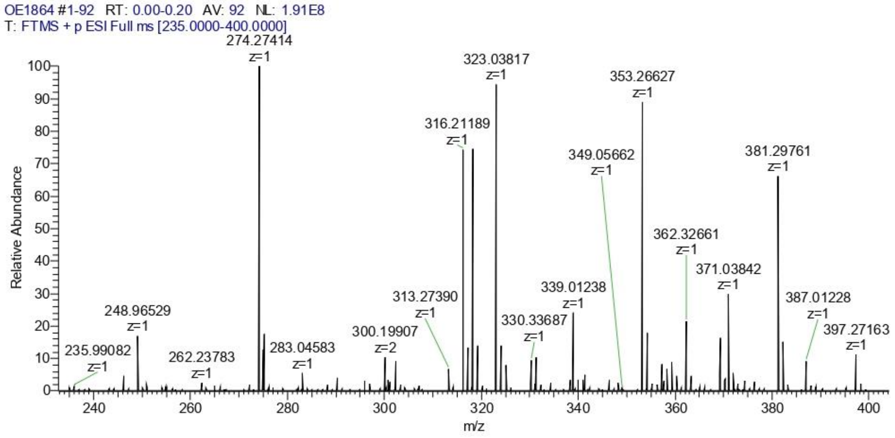

Zoomed spectra

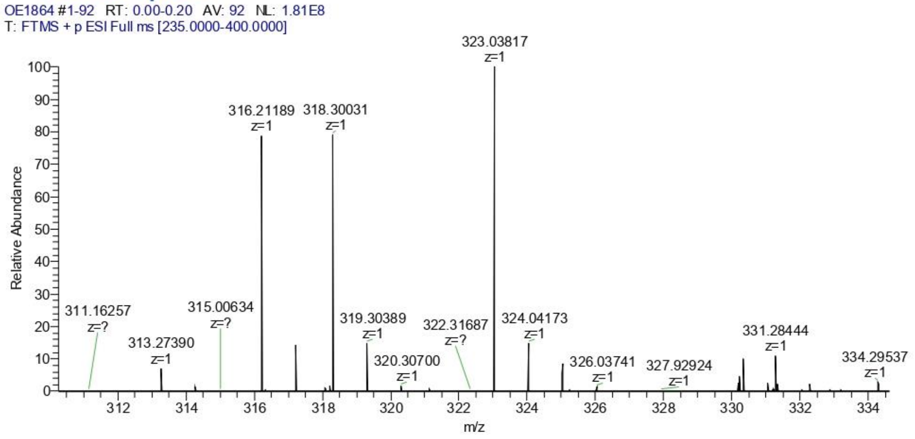

Exact mass	Observed mass	Observed ion type	Error (ppm)
323.03822	323.03817	$[M+Na]^+$	0.15

OE1866 PD(TS-3-TP)

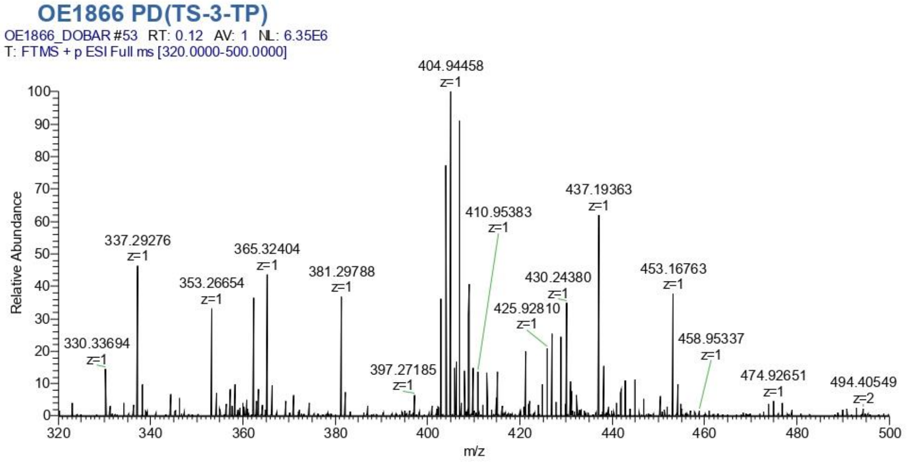

Zoomed spectra

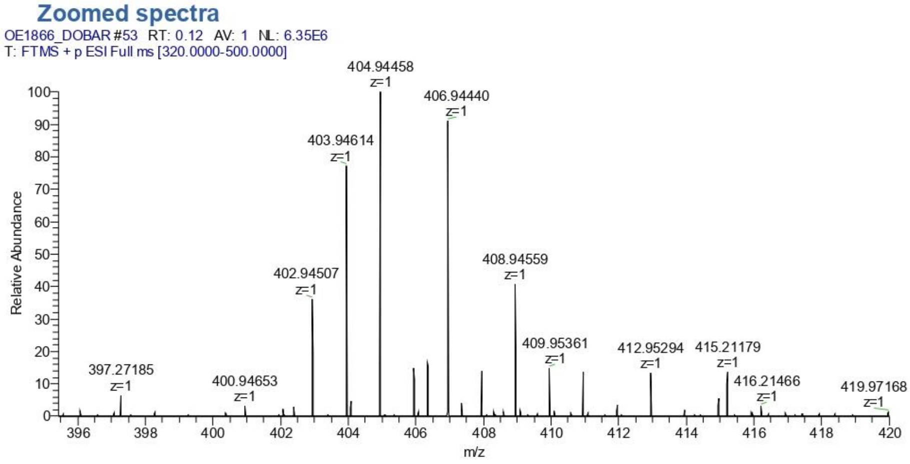

Exact mass	Observed mass	Observed ion type	Error (ppm)
404.94411	404.94458	$[M+H]^+$	1.16

OE1865 Cu(TS-3-TP)

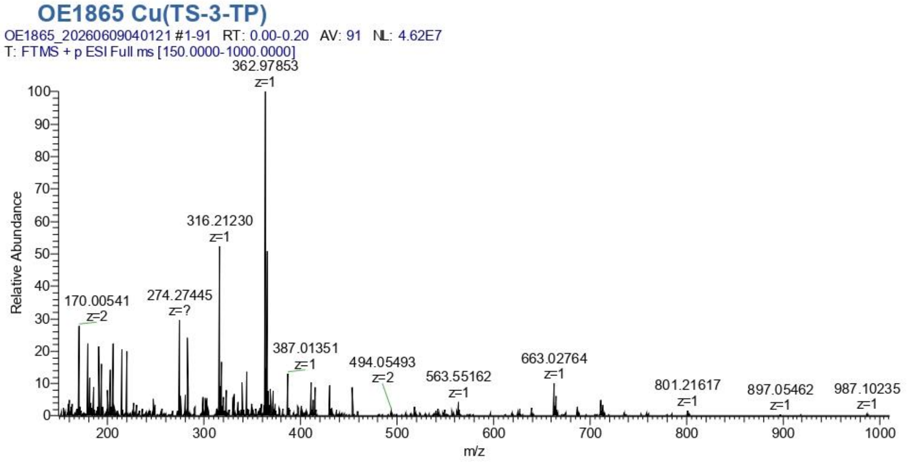

Zoomed spectra

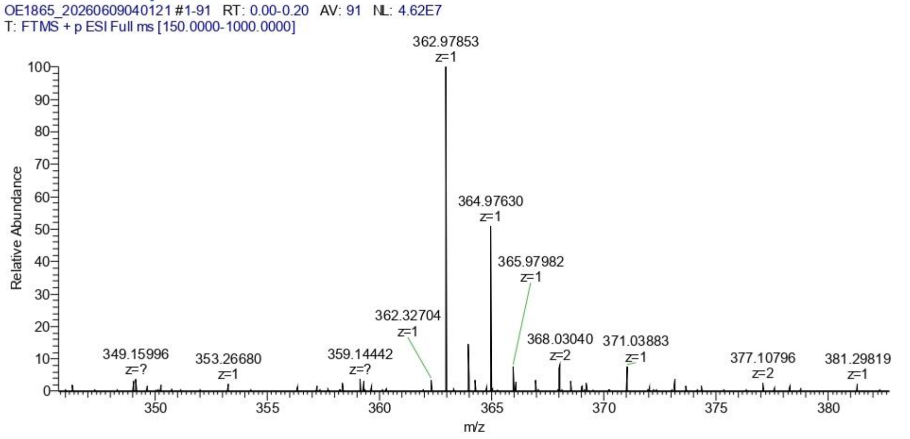

Exact mass	Observed mass	Observed ion type	Error (ppm)
362.97805	362.97853	$[C_{13}H_{16}O_4S_2Cu]^+$	0.00

Supplementary Figure S2

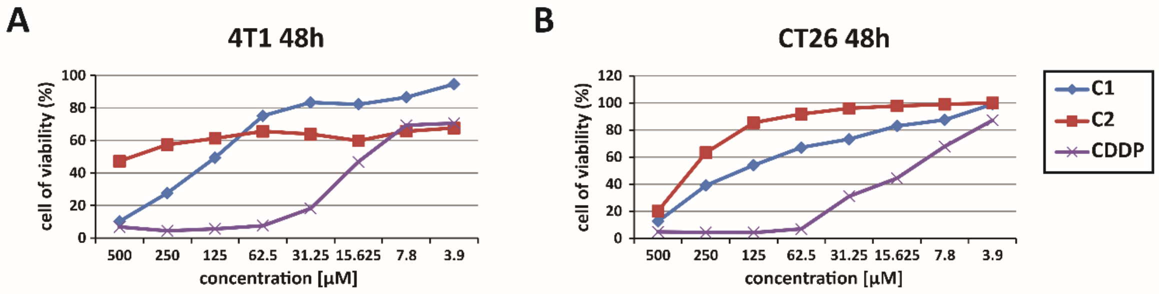

C

Cell line/complex	IC50 (µM) 48h C1	C2	CDDP
**4T1**	152.12±24.44	438.24±37.1	11.02 ±0.71
**CT26**	162.55±27.76	323.3±32.31	14.88 ±1.1

Supplementary Figure S3

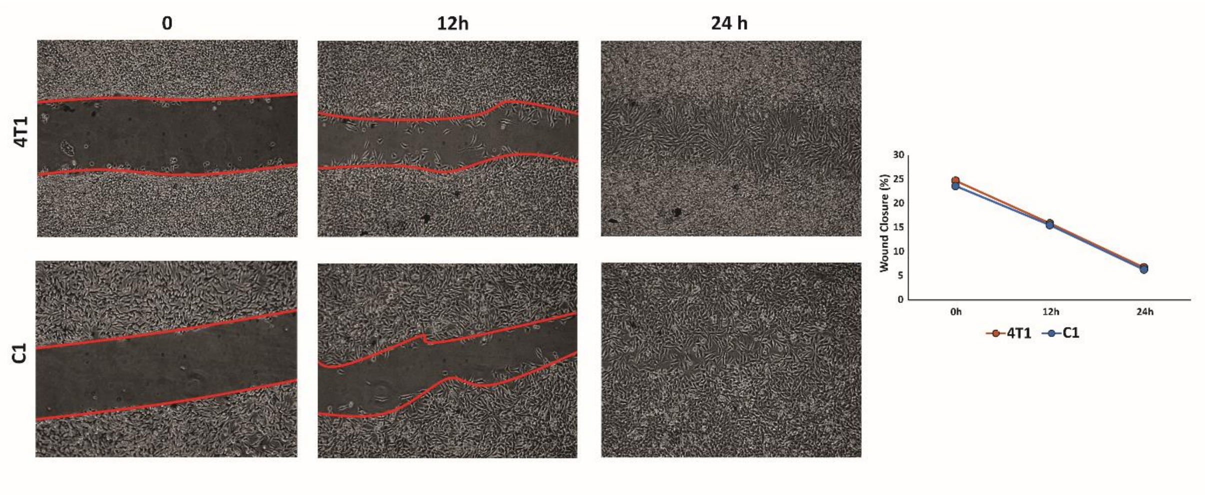

Supplement: Supplementary file 1 [file ijms-27-05659-s001.zip › ijms-4254790-supplementary.pdf]
